# Preimplantation chromosomal mosaics, chimaeras and confined placental mosaicism

**DOI:** 10.1530/RAF-21-0095

**Published:** 2022-04-05

**Authors:** John D West, Clare A Everett

**Affiliations:** 1 Section of Obstetrics and Gynaecology, Clinical Sciences, The Queen's Medical Research Institute, The University of Edinburgh, Edinburgh, UK

**Keywords:** chromosomal mosaic, chimaera, confined placental mosaicism, aneuploidy, mixoploidy

## Abstract

Some human preimplantation embryos are chromosomally mosaic. For technical reasons, estimates of the overall frequency vary widely from <15 to >90% and the true frequency remains unknown. Aneuploid/diploid and aneuploid/aneuploid mosaics typically arise during early cleavage stages before the embryonic genome is fully activated and when cell cycle checkpoints are not operating normally. Other mosaics include chaotic aneuploid mosaics and mixoploids, some of which arise by abnormal chromosome segregation at the first cleavage division. Chimaeras are similar to mosaics, in having two genetically distinct cell populations, but they arise from more than one zygote and occur less often. After implantation, the frequency of mosaic embryos declines to about 2% and most are trisomic/diploid mosaics, with trisomic cells confined to the placenta. Thus, few babies are born with chromosomal mosaicism. This review discusses the origin of different types of chromosomal mosaics and chimaeras; their fate and the relationship between preimplantation chromosomal mosaicism and confined placental mosaicism in human conceptuses and animal models. Abnormal cells in mosaic embryos may be depleted by cell death, other types of cell selection or cell correction but the most severely affected mosaic embryos probably die. Trisomic cells could become restricted to placental lineages if cell selection or correction is less effective in placental lineages and/or they are preferentially allocated to a placental lineage. However, the relationship between preimplantation mosaicism and confined placental mosaicism may be complex because the specific chromosome(s) involved will influence whether chromosomally abnormal cells survive predominately in the placental trophoblast and/or placental mesenchyme.

**Lay summary:**

Human cells normally have 23 pairs of chromosomes, which carry the genes. During the first few days of development, some human embryos are chromosomal mosaics. These mosaic embryos have both normal cells and cells with an abnormal number of chromosomes, which arise from the same fertilised egg. (More rarely, the different cell populations arise from more than one fertilised egg and these embryos are called chimaeras.) If chromosomally abnormal cells survive to term, they could cause birth defects. However, few abnormal cells survive and those that do are usually confined to the placenta, where they are less likely to cause harm. It is not yet understood how this restriction occurs but the type of chromosomal abnormality influences which placental tissues are affected. This review discusses the origin of different types of chromosomally abnormal cells, their fate and how they might become confined to the placenta in humans and animal models.

## Introduction

Mosaics are multicellular organisms composed of two or more genetically distinct cell populations that arise from a single zygote (Anderson *et al.* 1951, Ford 1969). Chromosomal mosaicism arises if a chromosomally different cell population is produced at any stage of development. Mosaicism may affect germ cells, somatic tissues or both and somatic mosaicism may be generalised or confined to a specific tissue or group of tissues. Generalised mosaicism is likely to arise early in development whereas confined mosaicism may arise at any stage. The clinical consequences of mosaicism for a somatic chromosomal abnormality depend on the abnormality; when and where it arises; the tissue distribution and the percentage of abnormal cells overall or in specific tissues (Spinner & Conlin 2014, Papavassiliou *et al.* 2015).

Chimaeras are similar to mosaics in having two genetically distinct cell populations but they arise in different ways. A chimaera was originally defined as ‘an organism whose cells derive from two or more distinct zygote lineages’ (Anderson *et al.* 1951). It has also been defined more broadly as ‘any composite animal or plant in which the different cell populations are derived from more than one fertilized egg or the union of more than two gametes” (McLaren 1976). This revised definition includes individuals where the two cell populations are from more than one zygote but not necessarily two whole zygotes.

Chimaeras are often classified as primary chimaeras, which arise very early in development, so all developmental lineages may be affected, or secondary chimaeras, which arise at postimplantation or postnatal stages (Ford 1969). Secondary chimaeras are beyond the scope of this review but include blood chimaeras, arising by placental fusion in dizygotic twin pregnancies (Dunsford *et al.* 1953); microchimaeras, which have a small number of donor cells from another individual that persist from bidirectional feto-maternal, cross-placental cell trafficking during pregnancy (Boddy *et al.* 2015) and those produced artificially by tissue transplantation.

Mosaics with numerical chromosome abnormalities contain either aneuploid or polyploid cells. Aneuploidy is when the number of chromosomes is not an exact multiple of the haploid number (n). It includes both chromosome gain (e.g. trisomy; Ts) and chromosome loss (e.g. monosomy; Ms) and may involve multiple chromosomes. Polyploid cells have an exact multiple of the haploid number of chromosomes but more than the diploid (2n) number. Polyploidy usually occurs as triploidy (3n; three haploid sets) or tetraploidy (4n; four haploid sets). Euploidy is when there is an exact multiple of the haploid number of chromosomes and includes haploidy, diploidy and polyploidy.

**Figure 1:**
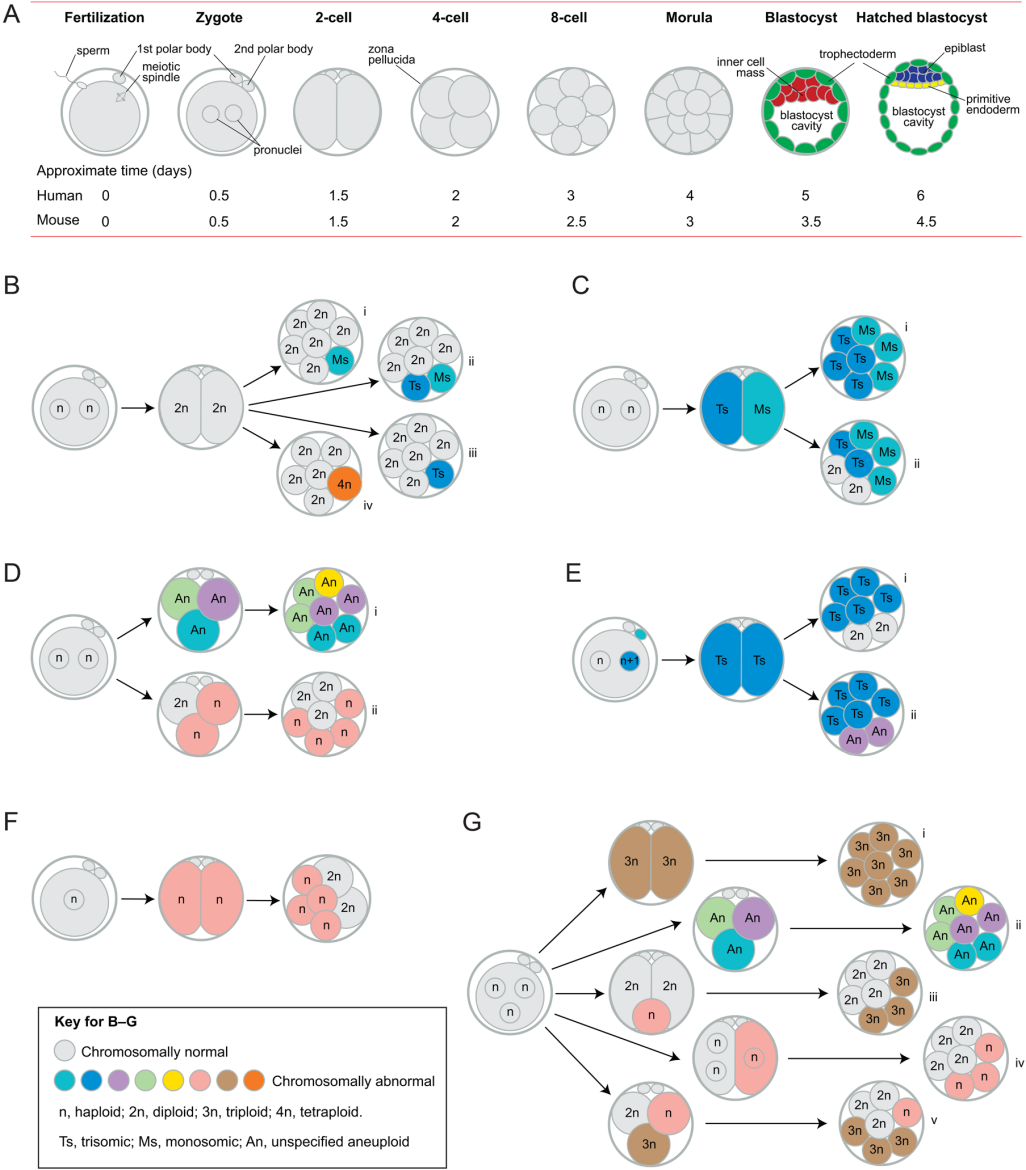
Preimplantation development, mosaicism and mixoploidy. (A) After fertilisation, the preimplantation embryo undergoes three cleavage divisions to the eight-cell stage (cleavage divisions are mitotic divisions that increase cell number but not embryo size and cells produced by cleavage division are called blastomeres). The embryo then compacts to form a morula, which cavitates to form a blastocyst, with an outer layer of trophectoderm cells surrounding the inner cell mass (ICM) and the blastocyst cavity. By the late blastocyst stage, the ICM forms the epiblast and primitive endoderm (hypoblast). (B) A normal diploid embryo may produce (i) monosomic/diploid, (ii) monosomic/trisomic/diploid, (iii) trisomic/diploid (origin may be indirect – see text) and (iv) tetraploid/diploid mosaics. (C) Non-disjunction, at the first mitotic division, would produce (i) monosomic/trisomic mosaics or (ii) other types of mosaics, including monosomic/trisomic/diploid mosaics, if further changes, such as trisomic rescue, occurred. (D) Normal bipronuclear zygotes sometimes form tripolar spindles and (i) individual chromosomes may segregate abnormally to produce a chaotic mosaic or (ii) entire haploid sets of chromosomes may segregate to form a haploid/diploid mixoploid. (E) A trisomic zygote could produce (i) trisomic/diploid or (ii) various types of trisomic/aneuploid mosaics. (F) A haploid zygote often produces some diploid cells by endoreplication, so forming a haploid/diploid mosaic. (G) A tripronuclear zygote may (i) produce a non-mosaic, triploid embryo; (ii) form a tripolar spindle and produce a chaotic mosaic which may continue to be unstable, (iii) extrude a small haploid cell which may fuse with a diploid cell to produce a triploid/diploid mixoploid, (iv) undergo atypical early cytokinesis so the pronuclei segregate passively to the two blastomeres or (v) form a tripolar spindle and segregate entire haploid sets of chromosomes to initially form a haploid/triploid/diploid mixoploid. See Fig. 2 for other details.

After fertilisation, the preimplantation embryo undergoes three cleavage divisions to the eight-cell stage, then compacts to form a morula, which cavitates to form a blastocyst, with an inner cell mass (ICM) and an outer layer of trophectoderm cells (Fig. 1A). The ICM divides into the epiblast and primitive endoderm (hypoblast) and the human blastocyst implants when it has about 256 cells (Niakan *et al.* 2012). Some human preimplantation embryos are chromosomal mosaics (Zenzes & Casper 1992, Delhanty *et al.* 1993) but estimates of their frequency vary widely from <15 to >90% due to the variety and limitations of techniques employed (discussed below) and the true frequency remains unknown. The occurrence of chromosomally mosaic preimplantation embryos has clinical implications for preimplantation genetic testing and this has been widely discussed elsewhere (Popovic *et al.* 2018).

There are substantially fewer chromosomal mosaics at postimplantation stages and about 2% of chorionic villus samples (CVS) are chromosomally mosaic (Wang *et al.* 1993, Pittalis *et al.* 1994, Huang *et al.* 2009, Malvestiti *et al.* 2015). Most of these mosaics occur as confined placental mosaicism (CPM) (Malvestiti *et al.* 2015), which is when chromosomally abnormal cells are present in the placenta but not in the fetus. This was first described by Kalousek and Dill (1983), although Warburton *et al.* (1978) had previously suggested its occurrence.

This review introduces different types of mosaic preimplantation embryos, involving whole chromosomes, and considers (i) how human preimplantation chromosomal mosaics and primary chimaeras arise; (ii) their fate; (iii) the relationship between preimplantation chromosomal mosaicism and CPM; (iv) some animal models of chromosomal mosaicism.

## Literature search

This is a narrative review of a broad topic and the authors decided which articles to include. Relevant articles were identified predominantly using Web of Science and PubMed to search the literature and the weekly PubCrawler alerting service to update them. Other relevant papers cited in these articles were also accessed. Search terms included ‘chimaera’, ‘chimera’, ‘chromosomal mosaic’, ‘confined placental mosaicism’, ‘mixoploidy’, ‘preimplantation genetic screening’ and ‘preimplantation genetic testing for aneuploidy’ in combination with other keywords relevant to the topic.

## Identification of mosaic preimplantation embryos

The extent of human preimplantation mosaicism only became apparent after the introduction of *in vitro* fertilisation (IVF). Initially, classical cytogenetics was used to determine the chromosomal constitution of preimplantation embryos (Angell *et al.* 1983) and an early review concluded that aneuploid/diploid mosaicism was the most common type of chromosomal abnormality (Zenzes & Casper 1992). However, the paucity of analysable metaphase spreads from preimplantation embryos made conventional cytogenetics inefficient so other methods were evaluated, including *in situ* hybridisation to specific chromosomes (Angell *et al.* 1987, West *et al.* 1987, Penketh *et al.* 1989). The introduction of multicolour fluorescence *in situ* hybridisation (FISH), to rapidly identify the copy number of several different chromosomes simultaneously in individual interphase cells of embryos, was a significant advance and provided important evidence for mosaicism among human preimplantation embryos (Griffin *et al.* 1992, Delhanty *et al.* 1993, Munné *et al.* 1993, Coonen *et al.* 1994, Munné *et al.* 1994).

FISH was improved to detect 8–12 different chromosomes and became widely used for preimplantation genetic screening for aneuploidy (PGS) for embryos produced by IVF. PGS was replaced by preimplantation genetic testing for aneuploidy (PGT-A) and FISH was superseded by several types of comprehensive chromosome screening (CCS) methods, which can analyse all 24 human chromosomes (Chen *et al.* 2020, Viotti 2020). CCS platforms that have been used for PGT-A include comparative genomic hybridisation (CGH) to metaphase chromosomes, array-CGH (aCGH), SNP microarrays, quantitative PCR and next-generation sequencing (NGS). NGS has some advantages and became widely used (Fiorentino *et al.* 2014). Additional powerful new techniques being used to investigate chromosomes in individual cells of embryos include evaluating haplotype and chromosome copy number by haplarithmisis (Destouni *et al.* 2016) and RNA-seq gene expression profiling (Starostik *et al.* 2020).

Estimates of the frequency of chromosomally mosaic preimplantation embryos vary. They depend on the detection method, numbers of cells and chromosomes analysed, the source of the cells and the criteria used to classify an embryo as a mosaic. Embryos rejected for transfer after IVF may be atypical and estimates based on a small biopsy may not be representative of the whole embryo. In some studies, an embryo is not classified as mosaic unless the proportion of abnormal cells reaches a specific threshold (van Echten-Arends *et al.* 2011). Also, blastocysts often contain tetraploid cells but studies differ as to whether they are counted as mosaic or normal.

An early 3–5-chromosome FISH study of embryos not selected for transfer reported 15.2% mosaics at the two to four-cell stage, 49.4% at five to eight cells and 58.1% by the morula stage (Bielanska *et al.* 2002). van Echten-Arends *et al.* (2011) reviewed 36 studies, comprising 815 whole embryos analysed by FISH or CCS, and reported mosaicism frequencies from 15 to >90% and a mean of 72% for cleavage stage embryos with at least eight chromosomes analysed. Overall, 22% were diploid, 59% were aneuploid/diploid mosaics, 14% were aneuploid/aneuploid mosaics and 5% had other numerical chromosomal abnormalities.

The high frequencies of mosaicism estimated by FISH are perhaps surprising, given that not all chromosomes are tested. FISH is less efficient with interphase nuclei than metaphase spreads (Ruangvutilert *et al.* 2000) and a theoretical model of the accuracy of 5-chromosome FISH predicted that it would overestimate the frequency of sporadic aneuploid cells and, therefore, the frequency of mosaicism (Scriven & Bossuyt 2010). Comparison of FISH and SNP-microarray analyses also suggested that FISH might overestimate the frequency of chromosomal mosaicism in cleavage stage embryos (Treff *et al.* 2010) although another study reported more consistent results for FISH, CGH and aCGH analyses of blastocysts (Fragouli *et al.* 2011). To what extent overestimation of mosaicism by FISH is offset by not testing all the chromosomes is unclear.

Marin *et al.* (2017) cited blastocyst mosaicism frequency estimates ranging from 4.8 to 44%, from NGS and other CCS studies of trophectoderm biopsies, with a typical value of approximately 15% for studies with multiple biopsies. An alternative method of estimating the frequency of mosaicism, based on the frequency of discordant CCS results after multiple biopsies, predicted 35.7% of blastocysts were mosaic, including 3.7% with reciprocal aneuploidies, from an analysis of 1124 embryos from 26 studies (Marin *et al.* 2021).

Starostik *et al.* (2020) analysed single-cell RNA-seq data from cells of 74 whole embryos on days 4–7 and estimated that, overall, 74% of morulae and blastocysts were mosaics with at least one cell affected by a mitotic error. However, the accuracy of RNA-seq analysis could be hampered by differences in gene expression among cells of early embryos and any differences in expression of maternal and paternal alleles.

Not all CCS platforms can detect mosaicism or polyploidy and results in the normal range are usually cited as euploid rather than diploid. When used in conjunction with SNPs, NGS can detect triploidy (Marin *et al.* 2018) but not tetraploidy, with equal numbers of maternal and paternal chromosomes. Although, in principle, NGS can detect mosaicism in PGT-A samples, this is not straightforward in a single trophectoderm biopsy of 5–10 cells because of the presence of two cell populations in a putative mosaic sample is inferred indirectly. NGS can produce accurate results with control mixtures of 5–10 aneuploid and diploid cells but is more robust with larger samples (Fragouli *et al.* 2017, Treff & Franasiak 2017, Biricik *et al.* 2021).

NGS produces different chromosome copy number profiles for monosomy, disomy and trisomy. Intermediate profiles are consistent with mosaicism but could also result from technical artefacts, so the frequency of mosaicism may be overestimated (Capalbo & Rienzi 2017, Capalbo *et al.* 2017). Conversely, however, other technical issues would cause the frequency of mosaics to be underestimated. One cell population present in a mosaic blastocyst may often be excluded from a small biopsy. Even if the biopsy is representative, some unbalanced mosaics, with a low proportion of one cell population, will be undetectable and mosaicism may be obscured if a biopsy contains both trisomic and monosomic cells of a reciprocal aneuploidy and is analysed as a single sample (Scott & Galliano 2016, Capalbo & Rienzi 2017, Treff & Franasiak 2017, Gleicher *et al.* 2021).

These technical limitations cast doubt on the reliability of mosaicism frequency estimates obtained by NGS analysis of small biopsies taken for PGT-A. Some authors suggest that this is likely to overestimate the frequency of mosaicism (Capalbo & Rienzi 2017, Capalbo *et al.* 2017, Marin *et al.* 2021, Treff & Marin 2021) but others consider that underestimation is more likely overall (Fragouli *et al.* 2017, Gleicher *et al.* 2021). Clinical implications of these technical limitations are discussed elsewhere (Capalbo *et al.* 2017, Paulson & Treff 2020, Gleicher *et al.* 2021, Treff & Marin 2021, Viotti *et al.* 2021*a*) and are beyond the scope of this review.

Although the combination of cytogenetics, FISH, NGS and RNA-seq analysis provides strong evidence that a substantial proportion of human preimplantation embryos are chromosomally mosaic, the true frequency remains unknown. It has been argued that mosaic preimplantation embryos may be significantly less common than was believed hitherto (Capalbo *et al.* 2017). This is based, in part, on four sets of data from human blastocysts (Fragouli *et al.* 2008, Johnson *et al.* 2010, Northrop *et al.* 2010, Capalbo *et al.* 2013), compiled by Capalbo and Rienzi (2017). The focus of this compilation was exclusively on the frequency of aneuploid/euploid mosaic blastocysts, which was estimated to be 6.1% (11/181). However, none of the four individual studies was designed specifically to determine the frequency of mosaicism in a typical cohort of human embryos and if other putative mosaics are included the overall estimated frequency of mosaicism is higher (~15%). Fragouli *et al.* (2011) also contrasted the total frequency of putative mosaic blastocysts in their study (33%; 17/52) with the lower frequency of putative aneuploid/diploid mosaics (17%; 9/52) and the much lower frequency of putative aneuploid/diploid mosaics with a majority of normal cells (5.8%; 3/52). Although aneuploid/diploid mosaicism may be most relevant to clinical PGT-A, all types of numerical chromosomal mosaicism should be included if the aim is to estimate the total biological frequency of mosaic preimplantation embryos.

Estimates of the total frequency of mosaic embryos are consistently substantially higher among preimplantation embryos than that reported for postimplantation stages. This is typically estimated as only about 2% of CVS (Wang *et al.* 1993, Pittalis *et al.* 1994, Huang *et al.* 2009, Malvestiti *et al.* 2015). Thus, for the purpose of this review, we make the conventional assumption that the frequency of mosaic embryos declines after implantation (Fragouli *et al.* 2013). Nevertheless, identification of the true frequency of preimplantation mosaicism would require identification of the chromosomal constitution of every cell from representative embryos at different stages, using an accurate, single-cell method for all the chromosomes.

As all human preimplantation embryos analysed for mosaicism are produced using assisted reproductive technology (ART), such as IVF or intracytoplasmic sperm injection (ICSI), it is not known whether a similar frequency of mosaicism occurs naturally. Some evidence suggests that the mosaicism frequency may be affected by ovarian stimulation protocols (Baart *et al.* 2007) and *in vitro* procedures (Swain 2019) and this is supported by several animal studies (discussed later). Nevertheless, postimplantation mosaicism frequencies are similar for natural pregnancies and those conceived by ART (Huang *et al.* 2009, Zamani Esteki *et al.* 2019).

## Origin of mosaic and chimaeric preimplantation embryos

Chromosomal mosaics are produced by postzygotic mitotic errors in either normal diploid embryos (Fig. 1B, C and D) or chromosomally abnormal embryos (Fig. 1E, F and G). One classification system distinguishes three broad categories: simple mosaics, produced by a single chromosomal error; complex mosaics; resulting from more than one error and chaotic mosaics, containing four or more chromosomally unrelated cell populations (Coonen *et al.* 2004). Chaotic mosaics may have no normal cells.

### Mosaics with aneuploid cells from diploid embryos

Most mitotic errors occur during the first three cleavage divisions (Munné *et al.* 1994, 2002), when development is still largely dependent on mRNA, protein and mitochondria inherited from the oocyte. (Major waves of human zygotic genome activation occur at 4–8 and 8–16 cells; Jukam *et al.* 2017.) This vulnerability may underlie why mitotic errors are so frequent during the early cleavage divisions. Inadequate stores of maternal transcripts and proteins could result in dysregulation of mitosis in early embryos, leading to segregation errors and aneuploid/diploid mosaicism (Mantikou *et al.* 2012).

Anaphase lag and non-disjunction are mitotic segregation errors that occur in early embryos and can arise from faults in cell cycle checkpoints (Vazquez-Diez *et al.* 2019), mitotic spindles (Chatzimeletiou *et al.* 2005) or cohesin proteins, which hold the two sister chromatids together (Mantikou *et al.* 2012). Cell cycle checkpoints G1/S, G2/M and the spindle assembly checkpoint (SAC or metaphase/anaphase checkpoint) each ensure that part of the cell cycle is completed properly before progressing to the next step. If an error occurs, the cell cycle is arrested until the problem is corrected or apoptosis is activated. If chromosomes misalign in early mouse embryos, the SAC components assemble but fail to inhibit the anaphase-promoting complex/cyclosome (APC/C), so anaphase progresses when it should be delayed (Vazquez-Diez *et al.* 2019). The authors suggest this could be caused by the altered stoichiometry of signalling components during the transition from maternal to embryonic control of gene expression. In human embryos, the SAC is also unable to activate apoptosis, to remove cells with malsegregating chromosomes, until the blastocyst stage (Jacobs *et al.* 2017).

Anaphase lag is when a chromatid fails to migrate with others at anaphase and can occur when a chromatid attaches to microtubules from both spindle poles or if sister chromatids separate prematurely (perhaps because of cohesin deficiency). The lagging chromatid fails to get incorporated into a nucleus and is often lost, producing an Ms/2n mosaic embryo (Fig. 1Bi). A chromatid can be sequestered into a micronucleus where it may become shattered and rearranged by chromothripsis (Pellestor *et al.* 2014), which is reminiscent of ‘chromosome demolition’, proposed by Los *et al.* (1998). Alternatively, the micronucleus may be passively inherited by one daughter cell or form an independent cellular fragment, which may fuse with an adjacent cell (Chavez *et al.* 2012, Vazquez-Diez *et al.* 2016, Vazquez-Diez & FitzHarris 2018).

Non-disjunction occurs when sister chromatids fail to separate (possibly from delayed cohesin removal; Mantikou *et al.* 2012), resulting in reciprocal aneuploidy with one trisomic and one monosomic cell. This produces Ms/Ts/2n mosaics (Fig. 1Bii) or Ms/Ts mosaics if non-disjunction occurs at the first cleavage division (Fig. 1C). Vazquez-Diez and FitzHarris (2018) suggested that anaphase lag could produce an equivalent outcome if a micronucleus, containing the lagging chromosome, is incorporated into a diploid nucleus.

It is unclear how simple Ts/2n mosaics could arise from diploid zygotes (Fig. 1Biii). Some authors have suggested that an individual chromosome may undergo endoreplication to produce a trisomic cell but this seems unlikely because endoreplication is expected to replicate the entire genome (Shu *et al.* 2018). Some Ts/2n mosaics could be secondary modifications of Ms/Ts/2n mosaics but monosomic cells may not die until apoptosis begins in the morula (Hardy *et al.* 2001). Ts/2n mosaics can also arise from trisomic zygotes (see below).

### Chaotic mosaics

Certain couples, including some with repeated implantation failure, are predisposed to producing complex or chaotic mosaic embryos (Fig. 1Di), suggesting a genetic basis (Delhanty *et al.* 1997, Mantzouratou *et al.* 2007, Voullaire *et al.* 2007). Mutations in genes affecting the SAC or chromatid-spindle attachment are implicated in a rare disease called mosaic variegated aneuploidy syndrome (Yost *et al.* 2017), which appears similar to complex or chaotic mosaicism in embryos. Comparable mutations might also affect embryos (Schmid *et al.* 2014), with only the mildest cases surviving postnatally. Evidence suggests that maternal inheritance of variants of the region of chromosome 4 that includes the candidate gene *PLK4* (polo-like kinase 4) may cause tripolar spindles in normally fertilised, bipronuclear zygotes, producing abnormal segregation of chromosomes to three daughter cells to form a chaotic mosaic (McCoy *et al.* 2015*b*, McCoy *et al.* 2018). PLK4 is required for regulating centriole duplication (Habedanck *et al.* 2005) and altered *PLK4* expression is associated with repeated implantation failure (Wang & Liu 2020). The production of chaotic mosaics from tripronuclear zygotes that form tripolar spindles is discussed below (Fig. 1Gii). In addition, chaotic mosaics may be produced by fertilisation with sperm retrieved from the seminal tract of some infertile men, particularly those with non-obstructive azoospermia (Magli *et al.* 2009).

### Mosaics from aneuploid embryos

Aneuploid embryos can also produce aneuploid/diploid or aneuploid/aneuploid mosaics (Fig. 1E). For example, a cell in a trisomic embryo may lose one copy of the trisomic chromosome by trisomic rescue (also known as ‘trisomic zygote rescue’), which could involve anaphase lag, non-disjunction or chromosome demolition (Kalousek *et al.* 1991, Los *et al.* 1998, Munné *et al.* 2005, Barbash-Hazan *et al.* 2009, Vazquez-Diez & FitzHarris 2018). However, unless chromosome loss was somehow targeted to the appropriate chromosome, it would produce new errors. Correction of a trisomic cell by mitotic anaphase lag would produce one corrected disomic cell and one trisomic cell, so a trisomic embryo would become a Ts/2n mosaic. Correction by mitotic non-disjunction would produce a disomic cell and a tetrasomic cell, which are likely to die. In one-third of cases, random loss of one copy of the trisomic chromosome would cause uniparental disomy (UPD, two maternal or paternal copies, instead of one of each). Depending on the chromosome involved, UPD can cause genomic imprinting abnormalities (Kotzot & Utermann 2005, Eggermann *et al.* 2015). Chromosome duplication for monosomic rescue could occur by non-disjunction (Conlin *et al.* 2010). This would always produce UPD and a nullisomic cell, which would die. However, although there is evidence for UPD from prenatal and postnatal samples (Eggermann *et al.* 2015), Gueye *et al.* (2014) only found UPD in 0.06% of human blastocysts. This suggests that aneuploid cell correction (aneuploid rescue) rarely occurs before implantation, even though mitotic anaphase lag and non-disjunction are likely to be most common during the cleavage stage. Such mitotic errors in aneuploid embryos would probably produce aneuploid/aneuploid mosaics more frequently than aneuploid/diploid mosaics.

### Mosaicism for segmental aneuploidy

Although the focus of this review is mosaicism for whole chromosome abnormalities, once CCS methods became available, losses and gains of chromosomal fragments, known as segmental (or partial) aneuploidy, were also identified in preimplantation embryos, either in all cells or in mosaic form (Voullaire *et al.* 2000, Wells & Delhanty 2000, Vanneste *et al.* 2009, Rabinowitz *et al.* 2012, Vera-Rodriguez *et al.* 2016, Babariya *et al.* 2017, Escribà *et al.* 2019). For example, Babariya *et al.* (2017) used aCGH to estimate that segmental aneuploidy occurred in 10.4% of human oocytes, 24.3% of cleavage stage embryos and 15.6% of blastocysts. Although some segmental errors may be inherited from a carrier of a balanced structural chromosomal abnormality and others may occur during meiosis, most arise during the early cleavage stages (Vera-Rodriguez *et al.* 2016, Babariya *et al.* 2017). Babariya *et al.* (2017) suggested that mosaicism for segmental aneuploidy could arise at cleavage stages if monitoring of DNA damage was dysfunctional before zygotic genome activation was completed and that suboptimal embryo culture conditions might also increase the frequency of double-stranded DNA breaks, leading to segmental aneuploidy. Different ways that segmental aneuploidy may arise are discussed elsewhere (Vanneste *et al.* 2009, Escribà *et al.* 2019).

### Mixoploidy

Different types of mixoploidy (combinations of polyploidy, haploidy and diploidy) can occur in preimplantation embryos, either alone or in combination with aneuploidy. Bielanska *et al.* (2002) analysed 216 preimplantation embryos at different stages and reported 2.8% haploid/diploid mixoploids (plus 0.5% haploids) and 14.8% polyploid/diploid mixoploids (plus 5.6% polyploids). The frequency of polyploid/diploid mixoploids was highest at the blastocyst stage (23/33; 69.7%) and most were tetraploid/diploid. Although the presence of tetraploid cells at cleavage stages may be abnormal, they are probably a normal feature of trophectoderm development in blastocysts (Angell *et al.* 1987, Benkhalifa *et al.* 1993, Bielanska *et al.* 2002, de Boer *et al.* 2004).

Some haploid/diploid mixoploids arise from haploid parthenote embryos, most of which generate diploid cells by the blastocyst stage (Fig. 1F; Leng *et al.* 2017). This occurs by failed cleavage, whereby the nucleus divides and the cleavage furrow forms but regresses, or endomitosis, a type of endoreplication whereby the nucleus divides without cleavage (Leng *et al.* 2017). Other haploid/diploid mixoploids may arise by non-canonical divisions that segregate complete haploid sets of chromosomes (Fig. 1Dii; Destouni *et al.* 2016), as discussed below. Tetraploid/diploid mixoploids may arise from diploid embryos (Fig. 1Biv) by endoreplication (Benkhalifa *et al.* 1993) or cell fusion (Balakier *et al.* 2000).

Approximately 2–9% of zygotes produced by conventional IVF are tripronuclear but only 14–25% of these, including most of those with an extra maternal pronucleus, divide into two triploid blastomeres (Fig. 1Gi; Angell *et al.* 1986, Kola *et al.* 1987, Pieters *et al.* 1992, Plachot & Crozet 1992, Palermo *et al.* 1994, Golubovsky 2003). There are more diandric triploids (extra paternal set of chromosomes) than digynic triploids (extra maternal set of chromosomes) among naturally conceived early abortuses (Zaragoza *et al.* 2000) and Plachot *et al.* (1989) found that 86% of preimplantation triploids, produced by conventional IVF, were diandric. However, Marin *et al.* (2018) reported that 5/5 ICSI-derived triploid blastocysts were digynic. Unlike conventional IVF, ICSI, with a single spermatozoon, precludes chromosomal abnormalities associated with polyspermy, such as diandric triploidy.

**Figure 2:**
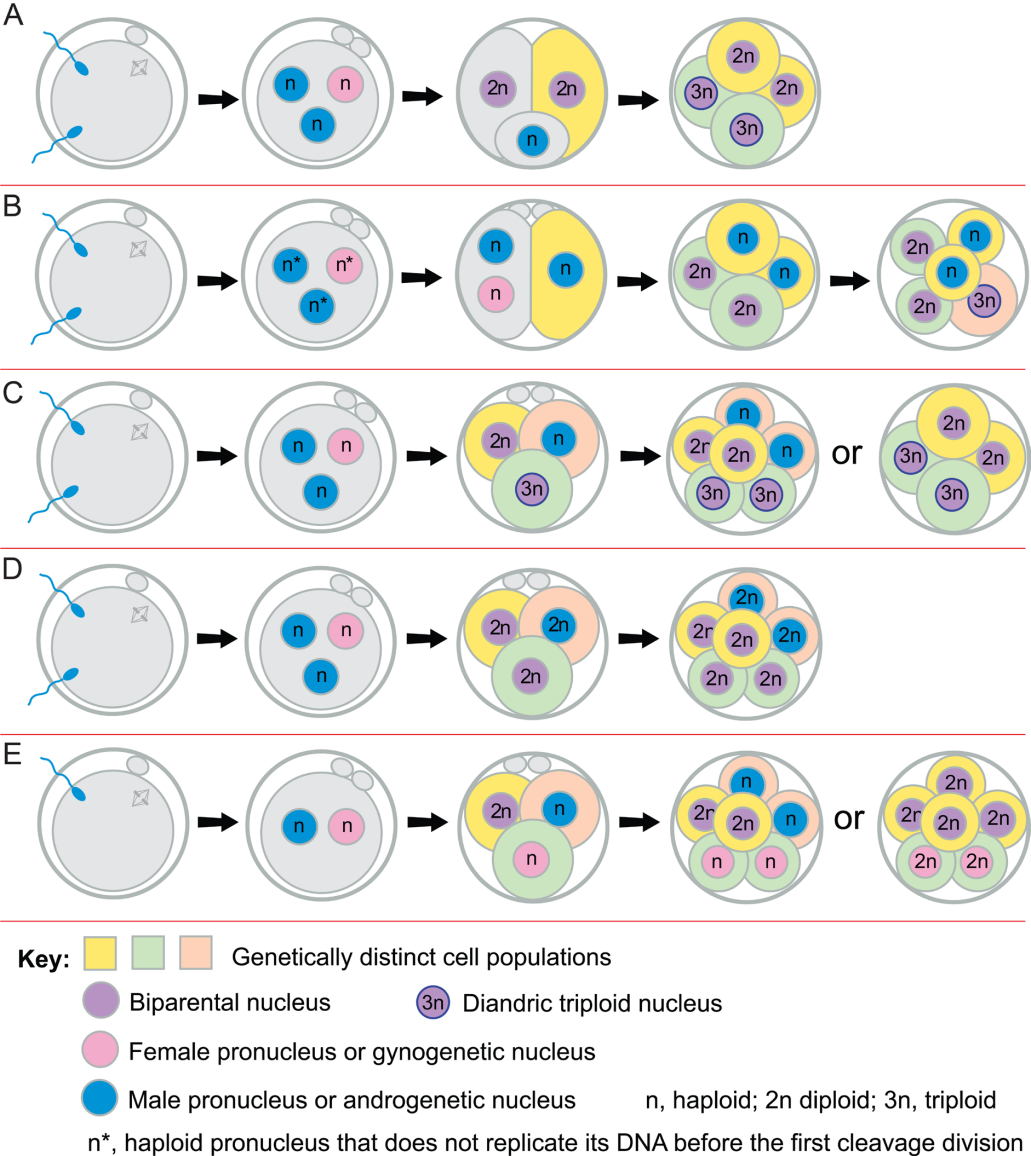
Segregation of complete haploid sets of parental chromosomes. (A, B, C and D) Three pronuclei form after dispermy and may segregate in different ways. (A) One male pronucleus is extruded in a small androgenetic haploid cell and the other two form two normal blastomeres. Later the extruded haploid cell may fuse with one blastomere and both blastomeres divide to form a triploid/diploid mixoploid embryo. (B) The zygote undergoes an atypical early cytokinesis and intact pronuclei are distributed between the two blastomeres. After the next division, there are two biparental diploid cells and two androgenetic haploid cells. In this example (from Supplementary Fig. 4A in Destouni *et al.* 2016) one diploid and one haploid cell fuse to produce a diandric triploid cell line and other cells divide but various fates are possible. Later the haploid cells may undergo endoreplication, fuse with other cells or die. (C) Entire haploid sets of chromosomes segregate on a tripolar spindle to form a mixoploid embryo with a biparental diploid, diandric triploid and an androgenetic haploid cell. Subsequently, haploid cells may die, fuse or endoreplicate to form androgenetic diploid cells or fuse with other cells. Only two of various possible fates are illustrated. (D) Entire haploid sets of chromosomes segregate on a tripolar spindle to form a chimaera with two biparental diploid cells with different paternal genomes and a diandric diploid cell with two different paternal sets of chromosomes. (E) Two pronuclei form after monospermic fertilisation but, occasionally, entire haploid sets of chromosomes may segregate on a tripolar spindle to form a chimaera with a biparental diploid cell, a gynogenetic haploid and an androgenetic haploid cell. Subsequently, haploid cells may die, endoreplicate to form diploid cells or fuse with other cells. Only two of various possible fates are illustrated.

Tripronuclear zygotes that arise by dispermic fertilisation have an extra centriole, as well as the extra paternal pronucleus, because centrioles are paternally inherited in humans (Palermo *et al.* 1994, Sathananthan *et al.* 1996). According to Golubovsky (2003) only about 25% of dispermic tripronuclear zygotes become triploid (Fig. 1Gi) and over 50% produce chaotic mosaic embryos because the supernumerary centriole produces a tripolar spindle and chromosomes segregate abnormally (Fig. 1Gii). Golubovsky (2003) further proposed that, in 14–32% of dispermic tripronuclear zygotes, one haploid pronucleus could separate and is either lost, leaving a diploid embryo, or retained, producing different types of mixoploids (Figs 1Giii-iv and 2A, B). Segregation of haploid and diploid sets of chromosome is supported by evidence that, in some tripronuclear zygotes, a complete haploid set of chromosomes is extruded (Fig. 2A) or segregates independently (Fig. 2B) to produce a haploid/diploid mixoploid, which may become 3n/2n by cell fusion and death of any remaining haploid cells (Angell *et al.* 1986, Kola *et al.* 1987, Pieters *et al.* 1992, Plachot & Crozet 1992, Golubovsky 2003, Rosenbusch & Schneider 2009). Mixoploids produced by this type of postzygotic diploidisation of triploids (Golubovsky 2003) could be considered to be chimaeras, by the broader definition given earlier.

More recent support for postzygotic diploidisation of triploids was obtained from single-cell haplotyping of tripronuclear bovine embryos by haplarithmisis. This showed that complete haploid, parental sets of chromosomes segregated independently at the first cleavage division in 9/23 (39%) of cleavage stage embryos produced by conventional IVF (Destouni *et al.* 2016) and this was termed ‘heterogoneic division’ (Greek for different parental origin). Destouni *et al.* (2016) found evidence for segregation of complete haploid sets of chromosomes in five embryos derived from tripronuclear zygotes, all of which had two paternal genomes, so were dispermic. They proposed that whole sets of chromosomes segregated on tripolar spindles and the chromosome complements could subsequently be modified if haploid cells died, diploidised or fused with other cells. Thus, dispermic, tripronuclear zygotes can produce mixoploidy (Figs 1Gv and 2C) or diploid/diploid chimaeras with androgenetic diploid and two types of biparental diploid cells (Fig. 2D). Destouni *et al.* (2016) also reported segregation of parental chromosome sets in two apparently normal monospermic, bipronuclear bovine zygotes. Thus, monospermic, bipronuclear zygotes can produce various types of chimaeras with different combinations of biparental, gynogenetic and androgenetic cells (Fig. 2E; Destouni *et al.* 2016, Destouni & Vermeesch 2017, Masset *et al.* 2021). Heterogoneic divisions might be facilitated if maternal and paternal chromosomes are kept apart on separate mitotic spindles during the first cleavage division in humans, as in mice (Reichmann *et al.* 2018), but further work is required to investigate this novel mechanism in humans.

### Other primary chimaeras and mosaics

Human chimaeras, including XX/XY chromosomal chimaeras, are much less common than mosaics and are usually only identified after birth, although many will remain undetected (particularly XX/XX and XY/XY chimaeras). Although primary chimaeras have less impact on assisted reproduction, it is appropriate to consider them alongside preimplantation mosaics because they arise at preimplantation stages. Molecular markers, for identifying the number of haploid genomes and their parental origin, have made it easier to distinguish chimaeras (with more than two haploid genomes) from mosaics and predict their possible mode of origin. Madan (2020) listed 50 human XX/XY (or similar) chimaeras (including six with an aneuploid cell line). These were identified because of abnormal sexual development, including hermaphroditism (28), congenital abnormalities (4), patchy skin (1) or by chance (17). Mosaic XX/XY individuals also occur because a 46,XX/46,XY mosaic may be produced from a 47,XXY zygote following two non-disjunction events (Niu *et al.* 2002).

**Figure 3:**
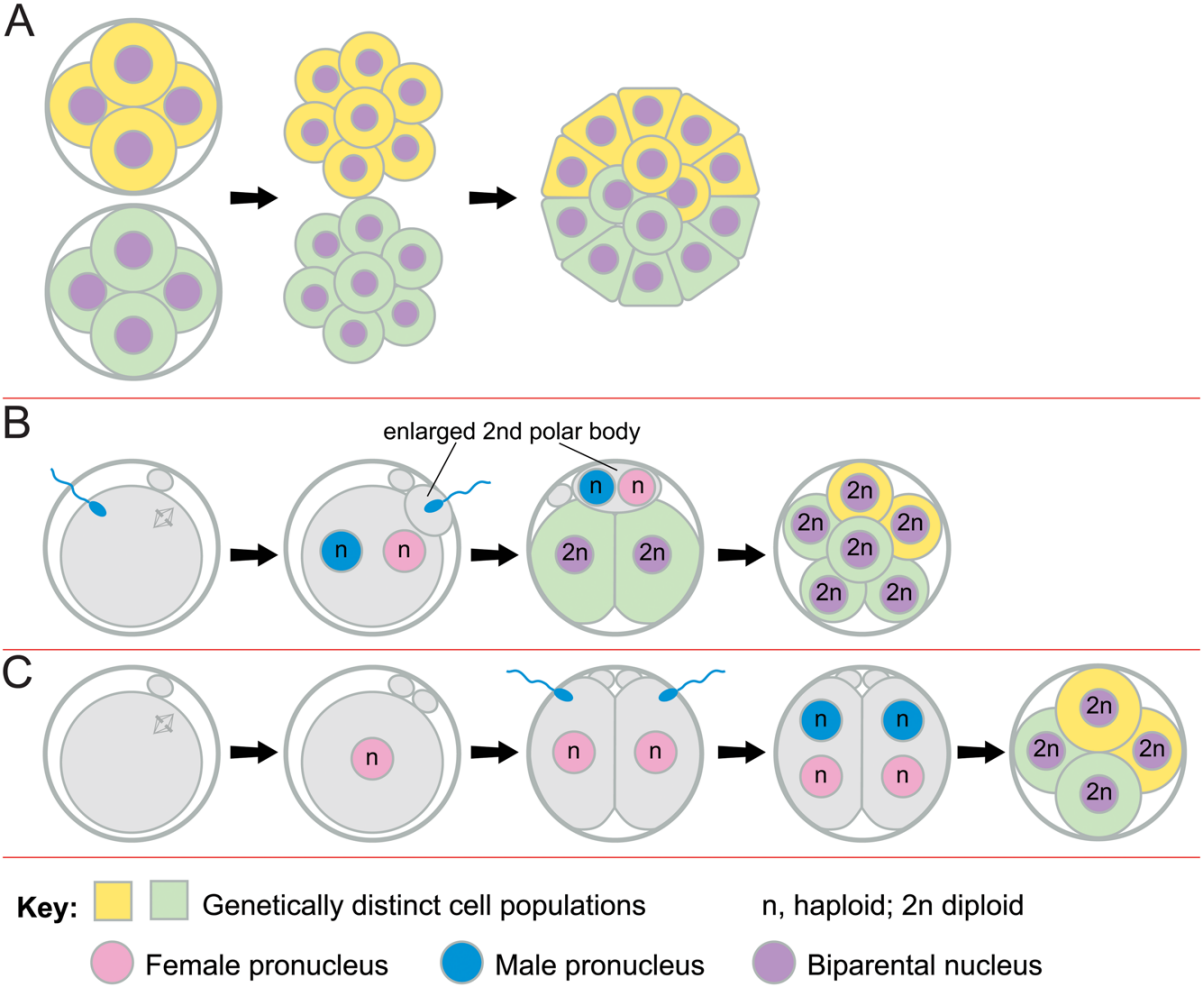
Different types of chimaeras. (A) Two cleavage stage embryos may lose their zonae pellucidae and aggregate to form an aggregation chimaera. (B) Fertilisation of both the egg and the second polar body, which may be enlarged. (C) Parthenogenetic activation of an oocyte produces a two-cell embryo with two haploid nuclei that are then both fertilised by different spermatozoa to produce a chimaera with identical maternal contributions in both cell lines.

A number of hypothetical mechanisms could produce primary chimaeras (Ford 1969, McLaren 1976) and Fig. 3 shows three that have been commonly invoked (Malan *et al.* 2006, Madan 2020). First, tetragametic aggregation chimaeras could occur if two preimplantation embryos aggregated after hatching from their zonae pellucidae (Fig. 3A). (The term ‘fusion chimaera’ is sometimes used but no cell fusion occurs.) Some aggregation chimaeras have been conceived in IVF programmes (Strain *et al.* 1998, Simon-Bouy *et al.* 2003), raising the possibility that IVF may facilitate embryo aggregation if multiple embryos are transferred to the uterus, particularly if assisted hatching is used. Late aggregation could result in two ICMs within one trophectoderm, which might produce chimaeric or non-chimaeric dizygotic twins within a single chorion (Souter *et al.* 2003, Miura & Niikawa 2005, Boklage 2006, Peters *et al.* 2017). Monochorionic dizygotic twins have been produced by IVF and most are chimaeric, at least in the blood (Peters *et al.* 2017).

Secondly, the egg and second polar body could be fertilised by different spermatozoa (Fig. 3B) and this might occur more readily if the polar body was enlarged, as reported for some aged mouse oocytes (McLaren 1976). Thirdly, diploid/diploid chimaeras, with two different paternal contributions but only one maternal contribution, could be produced if an oocyte is activated parthenogenetically and cleaves into two haploid blastomeres, which are then fertilised by different spermatozoa (Fig. 3C). Souter *et al.* (2007) reported twin chimaeras (one hermaphrodite and one male) that could have arisen this way if a chimaeric ICM divided to form twins, with different proportions of XX and XY cells.

Triploid/diploid mixoploidy may be produced in several ways other than those discussed earlier. These include aggregation of triploid and diploid embryos (Supplementary Fig. 1A, see section on supplementary materials given at the end of this article; Dewald *et al.* 1975, Daniel *et al.* 2003), delayed incorporation of an extra male pronucleus from a second spermatozoon (Supplementary Fig. 1B, C and D; Dewald *et al.* 1975, Daniel *et al.* 2003, Quigley *et al.* 2005) or delayed incorporation of an extra female pronucleus from the second polar body (Supplementary Fig. 1E and F; Ellis *et al.* 1963, Schmid & Vischer 1967, Müller *et al.* 1993, van de Laar *et al.* 2002, Daniel *et al.* 2003).

Chimaeras and mosaics with an androgenetic, gynogenetic or parthenogenetic diploid cell line plus a biparental diploid cell line may also be produced in several ways (Malan *et al.* 2006, Madan 2020) and can occur in combination with aneuploidy (Yamazawa *et al.* 2010). Examples shown in Supplementary Fig. 2 are from Surti *et al.* (2005) (Supplementary Fig. 2A), Kaiser-Rogers *et al.* (2006) (Supplementary Fig. 2B), Makrydimas *et al.* (2002) and Giurgea *et al.* (2006) (Supplementary Fig. 2C), Robinson *et al.* (2007) (Supplementary Fig. 2D and E), Sheppard *et al.* (2019) (Supplementary Fig. 2F) and Strain *et al.* (1995) (Supplementary Fig. 2G and H). As Destouni *et al.* (2016) pointed out, some of the types of chimaeras shown in Supplementary Figs 1 and 2 could also be produced by the segregation of complete parental sets of chromosomes at the first division (Fig. 2).

## Fate of preimplantation mosaic embryos

The fate of chromosomally abnormal cells in mosaics and chimaeras varies according to the type of abnormality. Haploid cells probably die, fuse with other cells or diploidise before implantation. As noted earlier, tetraploid cells commonly occur in blastocysts and they probably normally contribute to the placental trophoblast (Angell *et al.* 1987, Benkhalifa *et al.* 1993, Bielanska *et al.* 2002, de Boer *et al.* 2004). Triploid/diploid mixoploids can survive to fetal and postnatal stages (some with genomic imprinting disorders) but sometimes, triploid cells are confined to the placenta (Carson *et al.* 2018).

Most complex or chaotic mosaic aneuploid embryos die before implantation and some arrest before becoming blastocysts (Bielanska *et al.* 2002, Santos *et al.* 2010, McCoy *et al.* 2015*a*). The proportion of aneuploid cells in simple aneuploid/diploid mosaics declines between cleavage and blastocyst stages (Bielanska *et al.* 2002, van Echten-Arends *et al.* 2011, Fragouli *et al.* 2013). RNA-seq analysis also indicated that the proportion of aneuploid cells in mosaic embryos declined between days 3 and 6 (Yang *et al.* 2021) but that low-level mosaicism was still common among morulae and blastocysts, as noted earlier (Starostik *et al.* 2020). However, reports are contradictory as to whether the frequency of simple aneuploid/diploid mosaics increases or decreases by the blastocyst stage (van Echten-Arends *et al.* 2011, Fragouli *et al.* 2019).

Approximately 2% of CVS cases (Wang *et al.* 1993, Pittalis *et al.* 1994, Huang *et al.* 2009, Malvestiti *et al.* 2015), 0.25% of second-trimester amniocentesis samples (Hsu & Perlis 1984) and 0.02% of live births (Hassold & Jacobs 1984) are mosaic. Over 2700 embryos classified as putative mosaics have been transferred following PGT-A (reviewed by Treff & Marin 2021) but mosaicism has only been observed at term once (Kahraman *et al.* 2020). However, technical limitations mean that the classification of embryos as euploid, mosaic or aneuploid is inexact and make it difficult to determine the fate of mosaic embryos accurately (Marin *et al.* 2021, Treff & Marin 2021). Furthermore, chromosomal mosaicism may not be apparent at birth because postnatal cytogenetic investigations are not routine and are often confined to peripheral blood (Belva *et al.* 2020). Although the fate of mosaic embryos remains unproven, it seems likely that some mosaics, with a high proportion of aneuploid cells, miscarry and most others lose the aneuploid cells from the fetal lineage before term. As noted earlier, for the purpose of this review, we assume that the frequency of mosaicism is higher among preimplantation embryos and declines after implantation.

## Relationship between preimplantation chromosomal mosaicism and confined placental mosaicism

### Confined placental mosaicism

CPM is present in about 1–2% of viable human pregnancies tested by CVS, at approximately 10–12 weeks (Wang *et al.* 1993, Pittalis *et al.* 1994, Huang *et al.* 2009, Malvestiti *et al.* 2015). It is more common than true fetal mosaicism (TFM; Table 1) and often persists to term. Amniotic fluid cells, obtained by amniocentesis, are mostly from the fetus (Gosden 1983) and are used to distinguish between TFM and CPM. Different culture conditions for chorionic villus samples enrich the cytotrophoblast or mesenchymal cells and help identify three types of CPM, with abnormal cells in the villus cytotrophoblast (CPM-I), mesenchyme (CPM-II) or both (CPM-III). Studies differ as to whether CPM-I or CPM-II predominates (Pittalis *et al.* 1994, Malvestiti *et al.* 2015; Table 1).

**Table 1:** Frequencies of different types of mosaicism in two studies of chorionic villi and amniotic fluid cells.

Type of mosaic	Affected tissues			% of conceptuses		% of mosaics	
	Cytotrophoblast	Mesenchyme	AF cells	Study 1*	2*	1*	2*
Confined placental mosaicism							
CPM-I	Abnormal^†^	Normal	Normal	0.80	0.59	53.42	35.66
CPM-II	Normal	Abnormal	Normal	0.31	0.68	20.55	41.26
CPM-III	Abnormal	Abnormal	Normal	0.16	0.17	10.96	9.99
Total CPM				1.28	1.44	84.93	86.91
Fetal mosaicism							
TFM-IV	Abnormal	Normal	Abnormal	0	0.02	0	1.40
TFM-V	Normal	Abnormal	Abnormal	0.08	0.09	5.48	5.69
TFM-VI	Abnormal	Abnormal	Abnormal	0.12	0.10	8.22	5.99
CFM	Normal	Normal	Abnormal	0.02	–	1.37	–
Total FM				0.23	0.22	15.07	13.09

*Study 1 data are from Table 2 in Pittalis *et al.* (1994) and comprised 4,860 conceptuses, 73 of which were mosaics. Study 2 data are from Table 1 in Malvestiti *et al.* (2015) and comprised 60,347 conceptuses, 1001 of which were mosaics; ^†^Abnormal includes both uniformly abnormal and mosaic tissues.

AF, amniotic fluid. CFM, confined fetal mosaicism; CPM, confined placental mosaicism; FM, fetal mosaicism; TFM, true fetal mosaicism.

Only a subset of numerical chromosomal abnormalities found in preimplantation embryos is identified later at CVS. Most are autosomal trisomies or sex chromosome aneuploidies but triploidy (usually TFM) and tetraploidy (usually CPM) also occur (Pittalis *et al.* 1994, Malvestiti *et al.* 2015, Carson *et al.* 2018). Rare ‘confined placental chimaeras’, where a ‘vanishing twin’ contributes cells to the placenta, also occur (Falik-Borenstein *et al.* 1994, Surti *et al.* 2005). Different trisomies predominate in each of the three types of CPM (Lestou & Kalousek 1998, Benn & Grati 2021), which complicates investigations of CPM aetiology.

**Figure 4:**
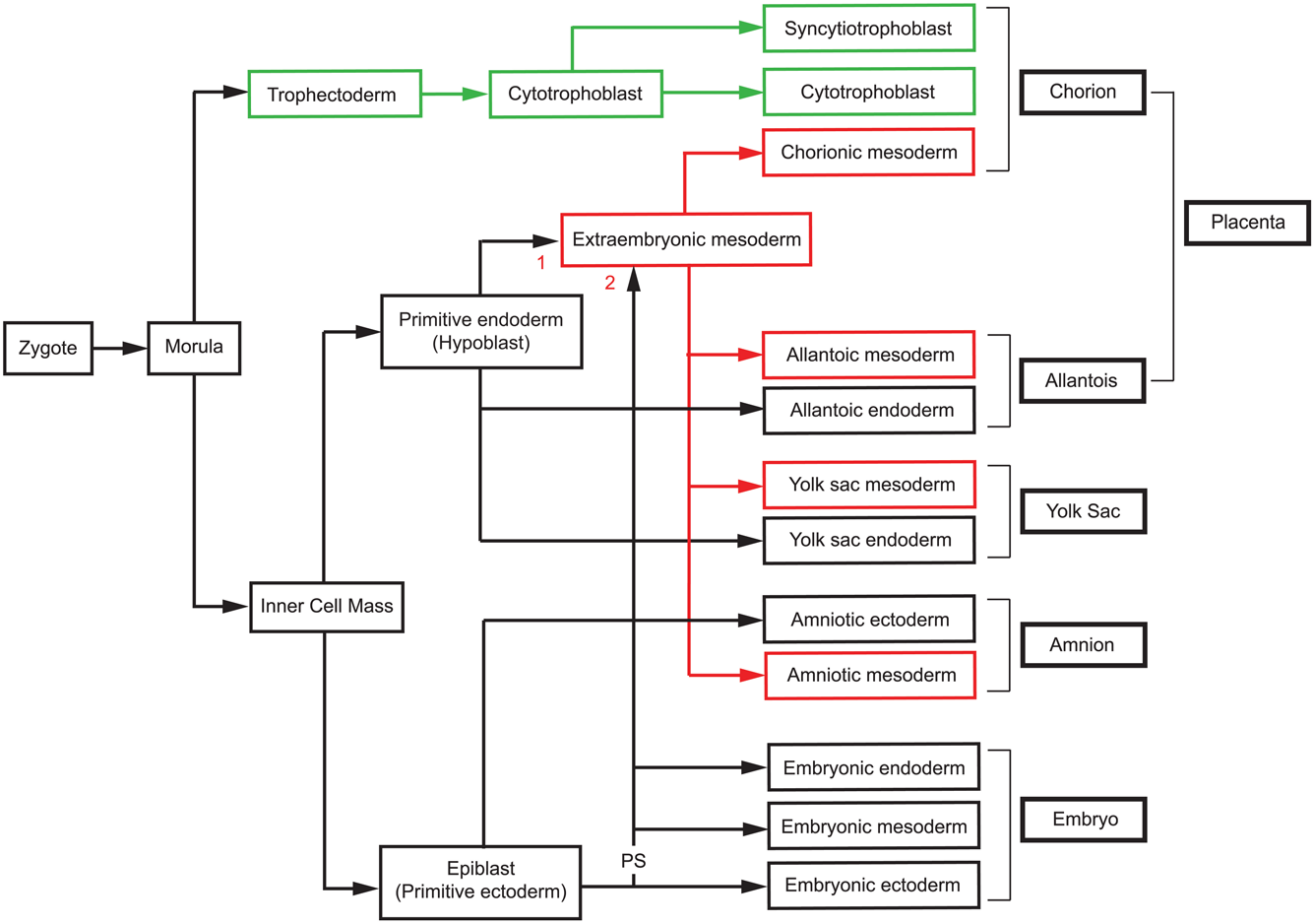
Simplified lineage diagram showing the origin of human extraembryonic tissues. The diagram shows the relationship between the embryonic lineage and the two lineages that produce the chorionic villi (trophectoderm and extraembryonic mesoderm). The three germ layers (ectoderm, mesoderm and endoderm) that form the embryo are produced from the epiblast and the embryonic mesoderm and embryonic endoderm emerge from the primitive streak (labelled ‘PS’) during gastrulation. Both the cytotrophoblast and syncytiotrophoblast are produced from the trophectoderm layer of the blastocyst. Evidence suggests that the human extraembryonic mesoderm is produced first by the primitive endoderm (labelled ‘1’) and later from epiblast via the primitive streak (labelled ‘2’). See text for references. For simplicity, both sources of cells are shown feeding into a common pool of extraembryonic mesoderm but they may colonise different tissues.

In CPM-III, abnormal (usually trisomic) cells are present in both the ICM and trophectoderm lineages, so they almost certainly exist before these two lineages segregate and are later excluded from the fetal lineage. In CPM-I, abnormal cells become restricted to the placental trophoblast whereas in CPM-II they become restricted to the placental mesenchymal core. This is produced by the extraembryonic mesoderm lineage, which appears to have a dual origin (Fig. 4). The first extraembryonic mesoderm appears before gastrulation and evidence implies this is derived from the primitive endoderm (hypoblast) lineage in both rhesus monkeys (Enders & King 1988) and humans (Bianchi *et al.* 1993). Later, additional extraembryonic mesoderm is produced from the epiblast lineage via the primitive streak at gastrulation (Enders & King 1988, Robinson *et al.* 2002).

The type of trisomic CPM is likely to depend on when, where and how (e.g. from a diploid or aneuploid zygote) the mosaicism arises, as well as the chromosome involved. Chance may also play a role because one cell population will be more easily excluded from a lineage founded by few cells and a minor cell population is likely to be more easily excluded. Robinson *et al.* (1997) proposed that CPM-I and CPM-II mainly arise from diploid zygotes (mitotic aneuploidy) whereas CPM-III is mainly from trisomic zygotes (meiotic aneuploidy) via trisomic rescue, which may cause UPD. CPM, with or without UPD, may affect fetal growth and development (Eggenhuizen *et al.* 2021).

In principle, four mechanisms could all contribute to the reduction of mosaic embryos and the predominance of CPM among surviving mosaics. These are (i) selective embryonic death (death of the most severely affected embryos), (ii) cell selection, (iii) cell correction and (iv) preferential allocation of abnormal cells to extraembryonic lineages. Preferential allocation would contribute to CPM, as would lineage-biased cell selection and/or lineage-biased cell correction if they were more effective in fetal than extraembryonic lineages. These four potential mechanisms for reducing the frequency of mosaic embryos are also discussed by Coticchio *et al.* (2021), using different terminology. We use the terms ‘selective embryonic death’ and ‘cell selection’ as previously (Everett & West 1996) and ‘cell correction’ whereas Coticchio *et al.* (2021) used ‘embryonic mortality’, ‘clonal depletion’ and ‘trisomic/monsomic rescue’, respectively.

### Selective embryonic death

As noted earlier, some mosaic embryos die before implantation. Approximately 1.1% of spontaneous abortions and 0.5% of stillbirths are Ts/2n mosaics (Hassold & Jacobs 1984). This suggests that selective embryonic death also occurs after implantation but it is not the main reason why the frequency of Ts/2n mosaics declines after implantation and few survive to term.

In a prospective, non-selection clinical trial, putative euploid and putative low-grade and medium-grade mosaic human blastocysts (estimated to have <50% aneuploid cells in the trophectoderm biopsy by NGS analysis) were made equally available for transfer and transferred to the uterus before NGS classifications were revealed (Capalbo *et al.* 2021). The putative mosaics were as likely to survive to term as putative euploid embryos but those with higher proportions of aneuploid cells were not transferred. Two retrospective studies suggested that putative mosaics with higher proportions of aneuploid cells and putative complex mosaics were less likely to survive (Munné *et al.* 2017, Viotti *et al.* 2021*b*). Although this is consistent with selective embryonic death of the most severely affected mosaics, the design of these studies has been criticised (Capalbo *et al.* 2021) and the difficulty of identifying true mosaic embryos from small biopsies was discussed earlier.

Some studies with animal models, described later, provide evidence for selective embryonic death and suggest that if too many abnormal cells persist in a postimplantation mosaic embryo it is more likely to die.

### Cell selection (clonal depletion)

Cell selection probably plays a major role in depleting the number of trisomic cells and restricting their distribution to the placenta but the degree of selection is likely to depend on the nature of the abnormality. Cell selection is thought to begin before implantation because, as noted earlier, the proportion of abnormal cells in mosaic embryos declines between cleavage and blastocyst stages (Bielanska *et al.* 2002, van Echten-Arends *et al.* 2011, Fragouli *et al.* 2013). Selection may involve cell death, loss and/or depletion, by reduced proliferation, but selection pressures are expected to be weak until relevant embryonic genes are activated, probably during the 4–16-cell period (Jukam *et al.* 2017). Possible mechanisms at preimplantation stages include apoptosis, which begins at the morula stage (Hardy *et al.* 2001), and exclusion of abnormal cells, particularly during morula compaction (Lagalla *et al.* 2017). Although direct evidence for selection against trisomic cells in human embryos is lacking, some cells excluded from human blastocysts are chromosomally abnormal (Orvieto *et al.* 2020). There is also some evidence for postnatal cell selection from longitudinal studies of trisomic/diploid mosaic children and adults but this may be more apparent in blood than tissues with a slower cell turnover (Gravholt *et al.* 1991, Papavassiliou *et al.* 2015). In embryos, cell selection against chromosomally abnormal cells might be more stringent in the epiblast or fetal lineage. For example, depletion of heterogeneous aneuploid cells by apoptosis occurred predominantly in the epiblast lineage of mouse chimaeras (Bolton *et al.* 2016) and the embryonic region of mosaic human ‘gastruloids’, produced with human embryonic stem cells (Yang *et al.* 2021). In these model systems, heterogeneous chromosomal mosaicism was induced experimentally and the mouse model is discussed later. DNA copy number variation analysis also suggests that the placental trophoblast may tolerate mutant or abnormal cells more readily than other tissues (Coorens *et al.* 2021).

### Cell correction

Aneuploid cells may be corrected by trisomic or monosomic rescue (discussed earlier), which can occur more than once in the same conceptus (Van Opstal *et al.* 2018). However, it is unclear whether this plays more than a minor role in restricting the distribution of aneuploid cells. As noted earlier, UPD is rare in human blastocysts (Gueye *et al.* 2014), suggesting that aneuploid cell correction rarely occurs before implantation, but UPD is more common in prenatal and postnatal samples (Eggermann *et al.* 2015). UPD might arise more frequently (per conceptus) after implantation, simply because many more mitotic divisions have occurred, providing more opportunities for cells with UPD to arise and proliferate. However, detection of a small proportion of UPD cells in a mosaic conceptus might still be challenging unless disomic cells have a selective advantage and outgrow the aneuploid cells.

### Preferential allocation to extraembryonic lineages

**Figure 5:**
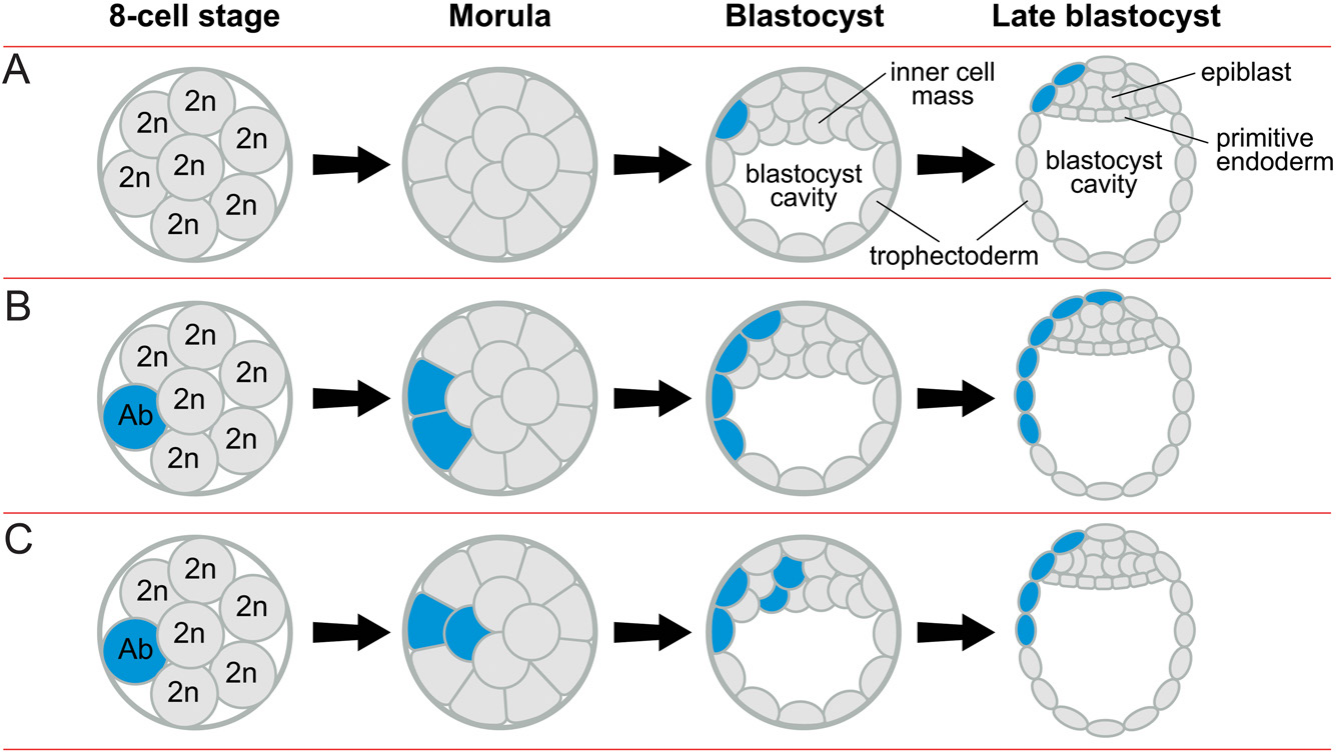
Events in preimplantation embryos that could cause or initiate CPM-I. By the late-blastocyst stage, there are three primary lineages, namely the epiblast, primitive endoderm (or hypoblast) and the trophectoderm, which produces all the placental trophoblast cells (Fig. 4). The outer morula cells form the trophectoderm layer and the inner cells form the ICM of the blastocyst. At least in the mouse, some outer morula cells produce additional inner cells by asymmetrical division and the epiblast and primitive endoderm (hypoblast) precursor cells are initially intermixed in the ICM but they assume their final positions in the late blastocyst (Saiz & Plusa 2013). In reality, restriction of abnormal cells to the trophectoderm/trophoblast lineage in CPM-I is likely to occur gradually and not be completed until after implantation. However, for simplicity, the figure illustrates how complete restriction of abnormal cells to the trophectoderm lineage could occur by the late blastocyst stage. (A) The chromosomally abnormal cell population (shaded blue) could arise exclusively in the trophectoderm at any time after it has separated from the ICM. (B) The abnormal cell population might arise at an early stage but be exclusively or predominantly allocated to the trophectoderm lineage. (C) The abnormal cell population could arise at an early stage and initially be present in both the ICM and trophectoderm but only survive in the trophectoderm lineage. Ab, chromosomally abnormal cell (shaded blue); 2n, normal diploid cell (shaded grey).

Preferential allocation of aneuploid cells to placental lineages at various stages could contribute to their restricted distribution. There is no convincing evidence for this in human embryos but studies have failed to allow for the observation that different trisomies predominate in CPM-I, CPM-II and CPM-III (Lestou & Kalousek 1998, Benn & Grati 2021). It, therefore, remains unknown whether any abnormal cell types are preferentially allocated to extraembryonic tissues. Most investigations have considered whether preferential allocation contributes to the restriction of aneuploid cells to the trophectoderm lineage in CPM-I. This restriction could occur if trisomic cells (i) arose preferentially in the trophectoderm or trophoblast, (ii) arose at the cleavage stage and were preferentially allocated to the trophectoderm or (iii) were present in multiple lineages initially but only survived in the trophectoderm/trophoblast (Fig. 5).

A recent NGS analysis of the distribution of putative aneuploid cells, among four trophectoderm samples and the ICM from dissected whole human blastocysts, indicated that they were often confined to one trophectoderm sample in putative aneuploid/diploid mosaics with low proportions of putative aneuploid cells (Capalbo *et al.* 2021). The authors suggested that aneuploid cells probably arose in the trophectoderm after it separated from the ICM, as shown in Fig. 5A. Aneuploid cells that arose earlier (Fig. 5B and C) might also sometimes produce spatially restricted cell populations if cell mixing was relatively limited before implantation, as in some mouse models (Garner & McLaren 1974, Gardner & Cockroft 1998, Everett *et al.* 2000).

However, restrictions could also begin later in development and/or involve multiple small steps. For example, abnormal cells might be preferentially (but not exclusively) allocated to the trophectoderm lineage at the blastocyst stage and abnormal cells remaining in other lineages lost after implantation. It is generally assumed that CPM develops from preimplantation mosaicism but, in some cases, trisomic cells may arise *de novo* in the placenta after implantation.

It is also important to consider how preferential allocation could result in CPM-II. If, the extraembryonic mesoderm of the placenta has a dual origin from the primitive endoderm and the epiblast (Fig. 4), CPM-II could arise if trisomic cells survived in the ICM, epiblast and/or primitive endoderm and later extraembryonic mesoderm lineages but became excluded from the trophectoderm/trophoblast and fetal lineages.

Most recent comparisons of the chromosomal composition of the ICM and trophectoderm have used comprehensive chromosome screening to analyse multicellular samples and categorised them as normal, mosaic or abnormal but none claimed a significant difference (Popovic *et al.* 2020). A more informative approach is to distinguish or separate ICM and trophectoderm cells and analyse each cell individually. Two such studies, using FISH, provided no evidence for preferential allocation of aneuploid cells to the trophectoderm (Evsikov & Verlinsky 1998, Derhaag *et al.* 2003). More recently, a study of gene expression profiles, to determine both cell type and chromosome content of individual cells, from day 4 morulae to day 7 blastocysts, showed no difference in the percentage of aneuploid trophectoderm and ICM cells (Starostik *et al.* 2020). However, aneuploid cells became enriched in the trophectoderm, relative to the epiblast and primitive endoderm, after culture to day 14, but there was no overall change in the aneuploidy frequency (Starostik *et al.* 2020). To unravel the relationship between preimplantation mosaicism and different types of CPM, it will be necessary to analyse results for different trisomies separately.

## Animal models of mosaicism

For ethical reasons, human embryo studies are largely descriptive, so animal models provide an important complementary approach. Indeed, many procedures used for human IVF and embryo culture are based on pioneering animal studies (Johnson 2019). Animal studies of chromosome segregation errors in preimplantation embryos are reviewed elsewhere (Vazquez-Diez & FitzHarris 2018) and some were mentioned earlier. A comprehensive discussion of animal models of chromosome mosaicism is beyond the scope of this review but some selected topics are considered.

### Preimplantation mosaicism *in vitro* and *in vivo*

Several animal studies suggest that IVF and/or embryo culture might increase mosaicism frequency. Two-colour FISH revealed a higher frequency of mosaicism among mouse blastocysts cultured from the two-cell stage than those developed *in vivo* (Sabhnani *et al.* 2011) and a higher frequency of sex-chromosome mosaicism when BALB/cWt strain mouse embryos were produced by IVF and cultured rather than developed *in vivo* (Bean *et al.* 2002).

Mixoploidy was reported to be more frequent when bovine, equine and ovine embryos were produced *in vitro* instead of *in vivo* (Viuff *et al.* 1999, Rambags *et al.* 2005, Coppola *et al.* 2007) and when bovine embryos were cultured *in vitro*, in the presence of serum, rather than developed in ewe oviducts *in vivo*(Lonergan *et al.* 2004). All four studies used two-colour FISH and reported the abnormal embryos as mixoploid but the scoring criteria precluded the identification of aneuploid/diploid mosaics. A more recent haplarithmisis study showed that both aneuploid and polyploid cells were more common among bovine embryos produced *in vitro* than *in vivo*(Tšuiko *et al.* 2017).

### Fate of chromosomally abnormal cells and animal models of CPM

Mice are commonly used to study the postimplantation fate of abnormal cells and lineage relationships are summarised in Supplementary Fig. 3. Early studies established that the mouse epiblast produces the fetus and extraembryonic mesoderm (including that of the amnion and yolk sac), the primitive endoderm produces extraembryonic endoderm, the polar trophectoderm forms placental trophoblast and the mural trophectoderm forms the trophoblast giant cells of the parietal yolk sac (Gardner & Papaioannou 1975, Gardner 1983). Later studies added complexity to the types of trophoblast giant cells (Simmons *et al.* 2007) and showed that the mouse primitive endoderm also contributes to the embryonic endoderm (Kwon *et al.* 2008) plus the allantoic and placental extraembryonic mesoderm (Rodriguez & Downs 2017).

#### Mixoploidy models

The fate of triploid cells has been studied in mouse chimaeras, produced either by aggregating cleavage stage triploid and diploid embryos or by fusing a haploid karyoplast or the second polar body to one cell at the two-cell stage. Triploid cells contributed more often to the trophectoderm than ICM in chimaeric blastocysts (Hino & Tateno 2016 and Azuma *et al*. 1991*b*, cited therein) but, in postimplantation chimaeras, they did not contribute consistently more to the placenta than the fetus and other epiblast derivatives (Everett *et al.* 2007, Hino & Tateno 2016). Instead, they contributed better to the yolk sac (Suwinska *et al.* 2005) or, specifically, the yolk sac endoderm (Everett *et al.* 2007). Triploid cells survived in fetal and adult tissues (Azuma *et al.* 1991*a*, Suwinska *et al.* 2005, Hino & Tateno 2016), although their proportion declined with fetal age (Suwinska *et al.* 2005), possibly because triploid cells divide relatively slowly (Hino & Tateno 2016).

Mouse tetraploid↔diploid (4n↔2n) aggregation chimaeras provided an early CPM model, following the observation that tetraploid cells were more abundant in extraembryonic tissues than the fetus of 4n/2n mouse mosaics (Tarkowski *et al.* 1977). In chimaeric blastocysts, tetraploid cells initially contributed to the epiblast, primitive endoderm and trophectoderm (Everett & West 1996). By embryonic day 12.5 (E12.5), however, they were excluded or severely depleted in the fetus and other epiblast derivatives but survived in the primitive endoderm and trophectoderm derivatives (James *et al.* 1995).

Further studies of mouse 4n↔2n chimaeras provided evidence for non-random allocation of tetraploid cells in chimaeric blastocysts, cell selection and selective embryonic death. Non-random cell allocation was suggested by enrichment for tetraploid cells in the trophectoderm, particularly the mural trophectoderm, which was reported in some studies of chimaeric blastocysts (Everett & West 1996, Tang *et al.* 2000, MacKay & West 2005) but not others (Ishiguro *et al.* 2005). Experimental manipulation showed that the larger size and increased ploidy of tetraploid cells could each affect their distribution in chimaeric blastocysts (Tang *et al.* 2000). In contrast, the initial tetraploid:diploid cell ratio had no consistent effect on cell distribution to different lineages (Tang & West 2000) but it affected the overall contribution of tetraploid cells (Goto *et al.* 2002).

Selection against tetraploid cells occurred during late preimplantation and postimplantation development (Everett & West 1998, Goto *et al.* 2002, Eakin *et al.* 2005, Ishiguro *et al.* 2005, MacKay & West 2005). Proliferation differences may contribute to cell selection (Eakin *et al.* 2005) but most tetraploid cells are probably eliminated from the epiblast lineage by a p53-dependent, tetraploid checkpoint that triggers apoptosis during epiblast differentiation (Horii *et al.* 2015). Although tetraploid cells have survived in some tissues of fetal and adult chimaeras (Lu & Markert 1980, Tarkowski *et al.* 2001, Goto *et al.* 2002), a high proportion may cause selective embryonic death (James *et al.* 1995, Tarkowski *et al.* 2005).

The trophectoderm of bovine embryos contained more putative polyploid cells than the embryonic disc on day 7 but the frequency declined significantly in both lineages by day 12 (Viuff *et al.* 2002). This provides evidence for cell selection but it is unclear whether the initial distribution resulted from non-random allocation or if more putative polyploid cells arose in the trophectoderm.

#### Models of mosaicism with aneuploidy

Mouse embryos have relatively low levels of aneuploid/diploid mosaicism but various models have been developed. Two knockout mouse models produce chaotic mosaics. *Sycp3^−/−^* female mice have a disrupted synaptonemal complex and produce aneuploid oocytes. About 30% of heterozygous *Sycp3^+/-^* embryos, with *Sycp3^−/−^* mothers, become chaotic mosaics, undergo p53-independent apoptosis, beginning in the embryonic ectoderm at E7.0, and die by E8.0 (Lightfoot *et al.* 2006). Homozygous *Bub1b^−/−^* embryos have a defective SAC, a high frequency of premature sister chromatid separation, become chaotic mosaics and die between E7.5 and E16.5 (Schmid *et al.* 2014).

Mouse embryos with non-specific aneuploidies were also produced by transient exposure to the SAC inhibitor, reversine, between the four-cell and eight-cell stages and provided evidence for lineage-biased cell selection and selective embryonic death (Bolton *et al.* 2016, Singla *et al.* 2020). Reversine-treated (RT), eight-cell embryos were aggregated with untreated, diploid embryos to produce RT↔diploid chimaeras. Cells from RT embryos (RT cells) were not preferentially allocated to the trophectoderm but were at a selective disadvantage from the blastocyst stage, predominantly in the ICM. This depletion continued after implantation and chimaeras with a high proportion of RT cells were more likely to die (selective embryonic death). Cell selection was mediated by elevated levels of apoptosis and autophagy in RT ICMs (particularly in epiblast cells) and a compensatory increase in proliferation of untreated cells. Apoptosis was less frequent in trophectoderm cells but RT trophectoderm cells divided more slowly than untreated trophectoderm cells by the sixth division. RT cells were not excluded significantly more frequently from the fetus than the placenta in postimplantation chimaeras and survived in some adults.

In the reversine treatment model, each cell of an eight-cell RT embryo can initiate an independent clone of abnormal cells and different clones may have multiple aneuploidies, single aneuploidies or no aneuploidy. Thus, there is heterogeneity among embryos and among cells within embryos. The ideal CPM model should produce simple, identifiable aneuploid/diploid mosaics. The fate of different aneuploidies should then be evaluated separately because some trisomies may model CPM-I and others may model CPM-II or CPM-III.

Mouse embryos with specific aneuploidies can be produced from stocks with Robertsonian translocations (Gropp *et al.* 1975) and other strategies have been used to produce XXY and XYY trisomic mice (Hirota *et al.* 2017). Partial trisomies of mouse chromosome 16 (Reeves *et al.* 1995, Sago *et al.* 1998) and a trisomic mouse strain carrying human chromosome 21 (O’Doherty *et al.* 2005) have also been created to model Down syndrome. Several specific aneuploidies have been incorporated into adult mouse chimeras and some were at a selective disadvantage (Epstein 1985). However, as far as we know, the contribution of a specific aneuploidy to the fetus and extraembryonic tissues has only been compared in two Ts3↔2n chimaeras, which did not show CPM (Everett *et al.* 2007).

Lightfoot *et al.* (2006) found that 20% of control, wild-type blastocysts were mosaic, suggesting that the frequency of spontaneous chromosomal mosaicism among mouse embryos might be higher than originally thought or vary among strains. Furthermore, culture (with or without IVF) would probably increase this frequency, as discussed earlier. Time-lapse video microscopy of cultured mouse embryos, created by IVF, showed that those with severe chromosome segregation abnormalities often formed micronuclei (Mashiko *et al.* 2020). This raises the possibility that, even without IVF, the use of time-lapse video microscopy to identify cultured embryos with micronuclei might provide a source of chromosomally mosaic embryos for experimental investigations, without using special stocks or inducing complex mosaicism.

### Some limitations of animal models

Care is required when extrapolating from animal models to humans. Mammalian karyotypes have been rearranged during evolution; so specific human aneuploidies have no exact equivalent in model species (Edwards 1994, Ferguson-Smith & Trifonov 2007).

The morphology of early postimplantation human and mouse embryos and the arrangement of the extraembryonic membranes differ (Supplementary Fig. 4) but this may be unimportant if the underlying developmental mechanisms are similar. Developmental differences among mammalian species were discussed by Eakin and Behringer (2004), who suggested that extraembryonic mesoderm has different origins in humans and mice. This would undermine the use of mice to model CPM-II but more recent evidence, discussed above, suggests that both the epiblast and primitive endoderm contribute to extraembryonic mesoderm in both species (Fig. 4 and Supplementary Fig. 3). Nevertheless, it remains unclear whether these different sources of extraembryonic mesoderm contribute comparably to human and mouse placental mesenchyme.

Zygotic genome activation occurs earlier in mouse embryos than in humans (Jukam *et al.* 2017), so cell cycle checkpoints would function earlier and this could contribute to their lower frequency of mosaicism. In humans and most mammalian species, except rodents, the centrioles are paternally inherited (Sutovsky & Schatten 2000) so dispermy delivers an extra centriole which often causes spindle abnormalities (Golubovsky 2003). However, this does not apply to mice.

Despite these limitations, more is known about the genetics and development of the mouse than other model species and much of this is likely to apply to humans. Undoubtedly, the mouse will continue to be an important model species for human chromosomal mosaicism but other animal models are also required.

## Conclusions

The true frequency of human preimplantation aneuploid/diploid mosaicism is unknown but most evidence suggests that it is quite common. Aneuploid/diploid mosaics typically arise during early cleavage stages, before the embryonic genome is activated and when cell cycle checkpoints are not fully functional. Abnormal chromosome segregation at the first cleavage division can also produce chaotic mosaics, mixoploids and some types of chimaeras but chimaeras probably occur relatively infrequently. The frequency of mosaics declines after implantation and mosaicism is usually confined to the placenta. To date, animal models have provided a number of insights into CPM but developmental differences between some animal models and humans need to be considered and might hinder progress. Further work, including separate analysis of different chromosomal abnormalities and the use of more refined animal models, is required to understand the relationship between preimplantation mosaicism and different types of CPM.

## Supplementary Material

Supplementary Material

## Declaration of interest

The authors declare that there is no conflict of interest that could be perceived as prejudicing the impartiality of this review.

## Funding

This work did not receive any specific grant from any funding agency in the public, commercial, or not-for-profit sector.

## Author contribution statement

J D W wrote the first complete draft of the manuscript; C A E wrote drafts of some sections. J D W and C A E reviewed the literature, prepared figures and reviewed and edited the final manuscript.
