# Supplementary material for: Preimplantation chromosomal mosaics, chimaeras and confined placental mosaicism
Source: Reprod Fertil. 2022 Apr 5;3(2):R66–90. doi: 10.1530/RAF-21-0095 (PMC9066951; doi:10.1530/RAF-21-0095)
Supplement: Supplementary Material [file supplementary_material.pdf]

Supplementary Material

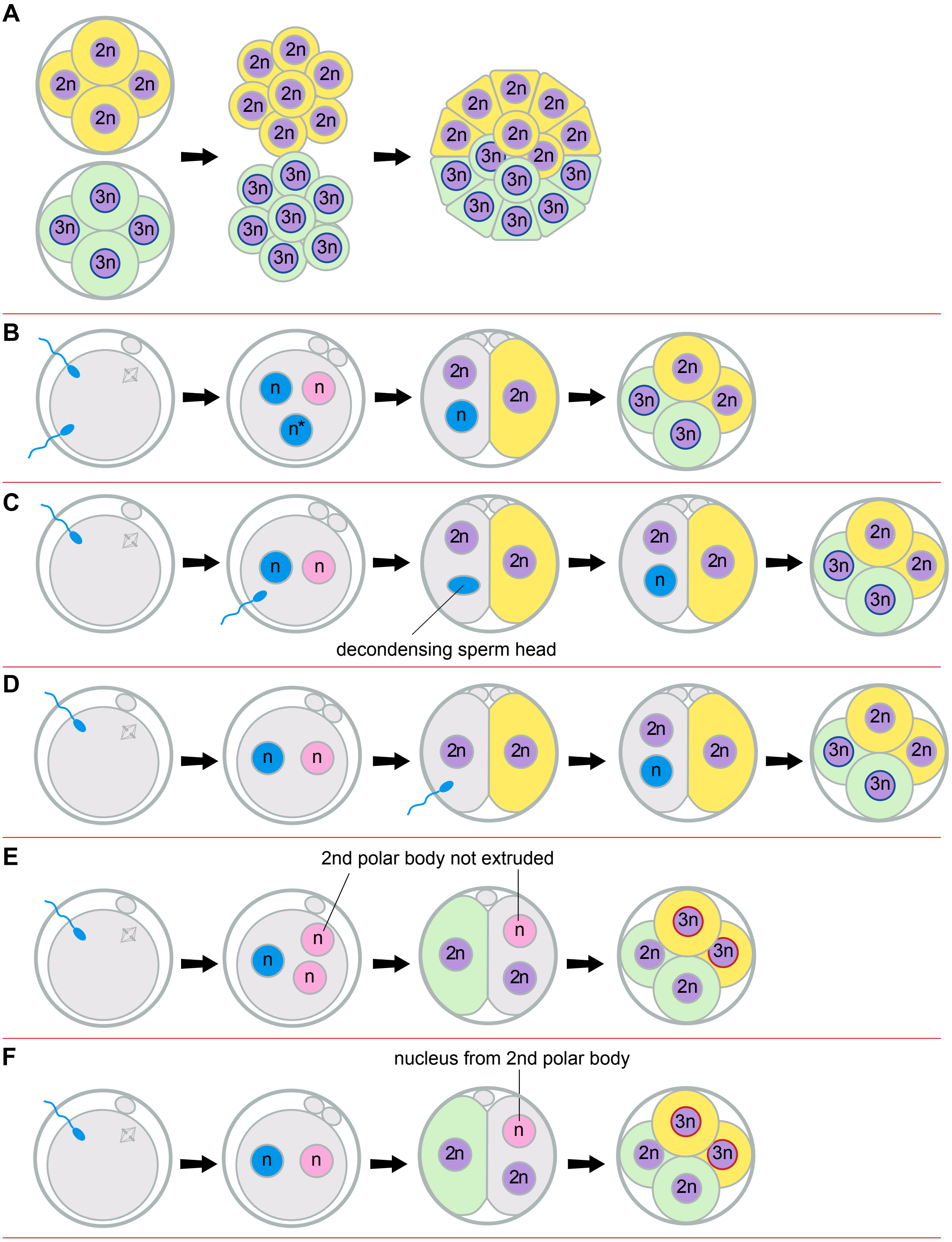

Key: Genetically distinct cell populations; Biparental nucleus

3n Digynic triploid nucleus; 3n Diandric triploid nucleus

Female pronucleus; Male pronucleus; n, haploid; 2n diploid; 3n, triploid

n*, haploid pronucleus that does not replicate its DNA before the first cleavage division

Fig. S1. Triploid/diploid mixoploids

Triploid/diploid mixoploidy may be produced in several ways other than those shown in Figs 1 and 2. **A.** Triploid↔diploid aggregation chimaera. **B-D.** Dispermy and late incorporation of one male pronucleus into only one cell at the 2-cell stage results in diandric triploid/diploid mixoploidy. (B) One pronucleus fails to replicate its DNA before the first cleavage division and is inherited passively by one daughter cell, which becomes triploid. (C) Entry of the second spermatozoon is delayed, so the first cleavage occurs before the sperm head is fully decondensed and the male pronucleus is restricted to one cell. (D) Entry of the second spermatozoon is delayed until after the first cleavage division, so it enters only one cell. **E,F.** Digynic triploid/diploid mixoploidy from late incorporation of an extra female pronucleus from the second polar body to one cell at the 2-cell stage to create a triploid cell line. (E) The second polar body is not extruded and the extra female pronucleus passes passively to one cell. (F) The second polar body fuses with one of the cells.

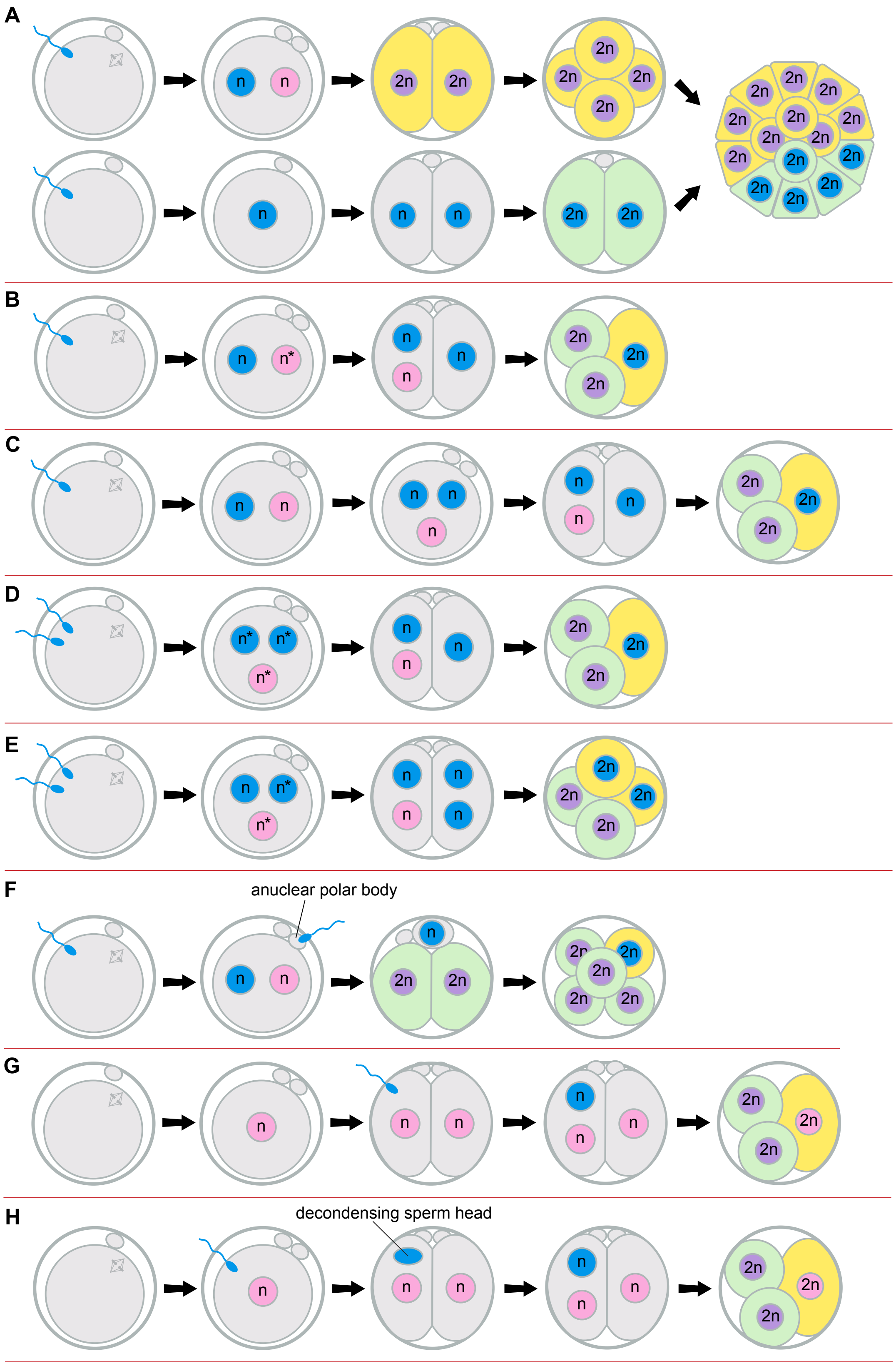

Key: Genetically distinct cell populations — Biparental nucleus

Female pronucleus or parthenogenetic or gynogenetic nucleus

Male pronucleus or androgenetic nucleus — n, haploid; 2n diploid

n*, haploid pronucleus that does not replicate its DNA before the first cleavage division

Fig. S2. Androgenetic and parthenogenetic chimaeras and mosaics

Several hypotheses have been proposed to account for individuals with both a biparental diploid cell line and an androgenetic or parthenogenetic diploid cell line. Some would arise from a single zygote, so would be considered to be mosaics, but others would arise from more than one zygote, so would be considered to be chimaeras according to some definitions. **A.** Spontaneous aggregation of a biparental diploid embryo and an androgenetic diploid embryo to produce a chimaera (Surti et al., 2005). The androgenetic zygote could be produced by fertilization of an empty oocyte with no functional meiotic spindle. This would initially be haploid but it could diploidize by endoreplication during the first few cleavage divisions. (The fertilizing spermatozoon would have to carry the X chromosome because 46,YY embryos would die.) In principle, chimaeras could also be formed by aggregation of parthenogenetic and biparental diploid embryos (not illustrated). **B-F.** Other embryos with a biparental diploid cell line and an androgenetic diploid cell line. **B.** The oocyte is fertilized normally and the male pronucleus replicates its genome and undergoes a normal first mitotic division but the female pronucleus fails to replicate its genome before the zygote cleaves. Its nuclear membrane remains intact and it is inherited passively by one daughter cell. Thus, the 2-cell stage embryo has one cell with two haploid nuclei and one cell with one haploid androgenetic nucleus, which diploidizes by endoreplication. At later stages the embryo has both normal biparental diploid and androgenetic diploid cells (Kaiser-Rogers et al., 2006). **C.** An alternative possibility is that the paternal pronucleus replicates to produce a third pronucleus in the zygote. At the first cleavage division, these three haploid genomes are passively allocated to the two daughter cells to produce one normal diploid cell and one haploid androgenetic cell, which would then endoreplicate to become diploid (Makrydimas et al., 2002; Giurgea et al., 2006). (If the three haploid nuclei are distributed randomly to two cells, other genotype combinations could also be produced, including androgenetic/gynogenetic embryos.) **D.** After dispermic fertilization three pronuclei form but do not replicate their DNA. The zygote cleaves and the intact pronuclei are distributed randomly to the two cells and replicate their DNA prior to the next division. At the 2-cell stage, in the example illustrated, one cell with one male and one female pronucleus divides to produce two biparental diploid cells. The haploid nucleus in the other cell, at the 2-cell stage, may undergo endoreplication and produce an androgenetic diploid cell line (Robinson et al., 2007). (As in (C), if the three haploid nuclei are distributed randomly to two cells, other genotype combinations could also be produced.) **E.** Alternatively, after dispermic fertilization, two male and one female pronuclei form but only one male pronucleus replicates its DNA. This pronucleus undergoes a normal first mitotic division but the other two pronuclei fail to replicate their genomes and are inherited passively. At the 2-cell stage, in the example illustrated, one cell, with one male and one female pronucleus, divides to produce a biparental diploid cell line. The other cell, at the 2-cell stage, has two male pronuclei and divides to produce an androgenetic diploid cell line (Robinson et al., 2007). **F.** Separate fertilization of the egg and an anuclear polar body, which endoreplicates to produce an androgenetic diploid cell line (Sheppard et al., 2019). **G,H.** Embryos with both a biparental diploid cell line and parthenogenetic diploid cell line may be produced if parthenogenetic activation of an oocyte is followed by delayed fertilization, so the spermatozoon only contributes to one cell at the two-cell stage. This cell produces a biparental diploid cell line. The haploid cell diploidizes by endoreplication and forms a parthenogenetic diploid cell line. The authors suggest two possible mechanisms and prefer (H) over (G) on biological grounds (Strain et al., 1995). In (G), entry of the spermatozoon is delayed until after the first cleavage division so it enters only one cell. In (H) the spermatozoon enters before the first cleavage division but is delayed so the sperm head has not fully decondensed when cleavage occurs and the male pronucleus is restricted to one cell.

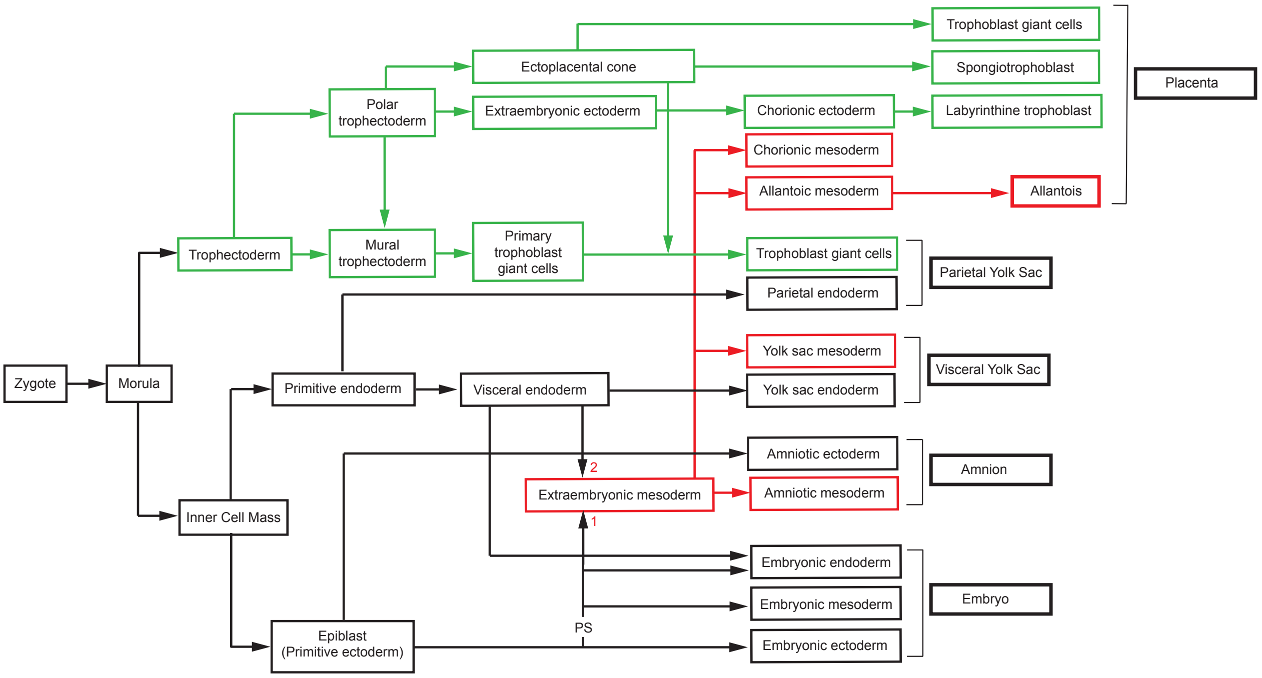

Fig. S3. Simplified lineage diagram showing origin of mouse extraembryonic tissues

The diagram shows the relationship between the embryonic lineage and the two lineages that produce the placenta (trophectoderm and extraembryonic mesoderm). There are multiple types of trophoblast giant cells in the mouse placenta and some may be produced by the extraembryonic ectoderm lineage but for simplicity this is not shown. The three germ layers (ectoderm, mesoderm and endoderm) that form the embryo are produced from the epiblast. The embryonic mesoderm and embryonic endoderm emerge from the primitive streak (labelled "PS") during gastrulation. It is well established that mouse extraembryonic mesoderm is produced by the epiblast via the primitive streak (labelled "1"). However, more recent evidence shows that the visceral endoderm also contributes to the placental extraembryonic mesoderm (labelled "2") and the embryonic endoderm. For simplicity, two sources of cells are shown feeding into a common pool of extraembryonic mesoderm but they may colonise different tissues. See text for references.

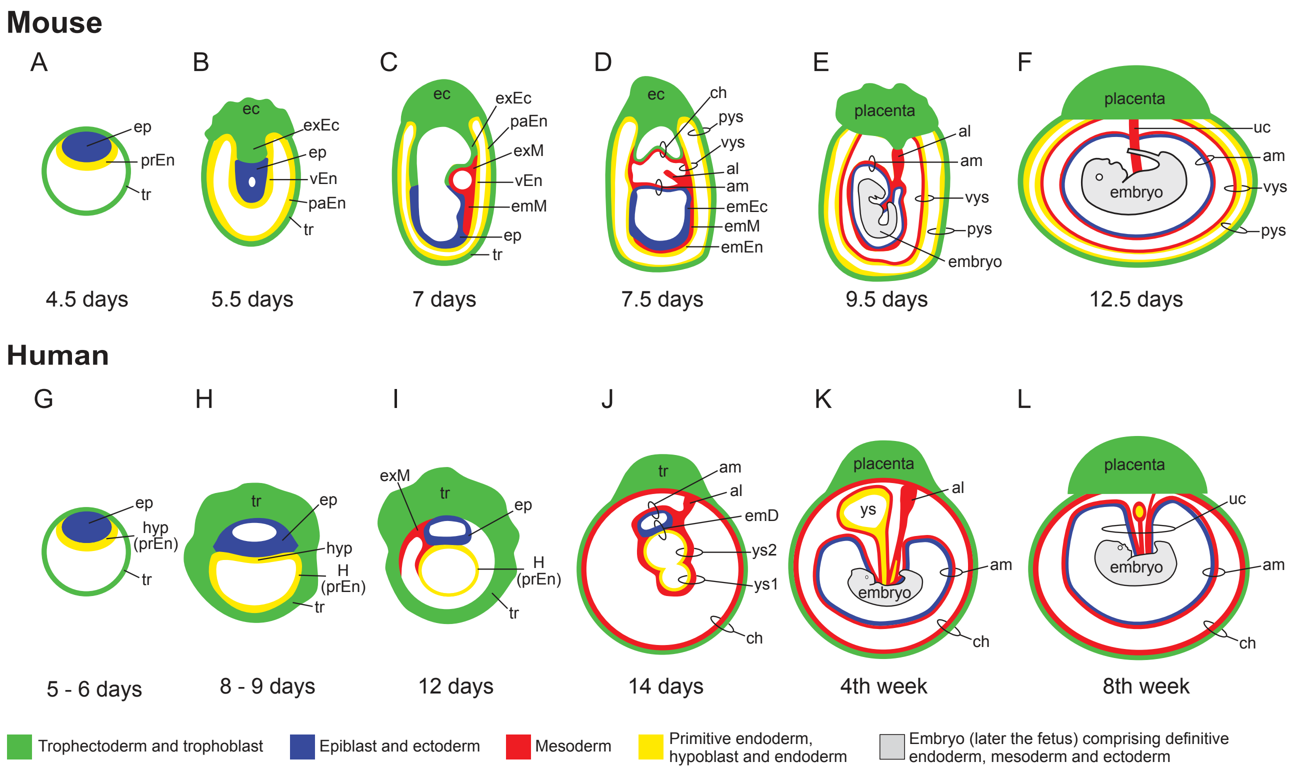

Fig. S4. Comparison of extraembryonic development in the mouse and human

Simplified comparison of the morphology of the extraembryonic membranes during development of the mouse and human conceptuses. The blastocyst stages in the mouse (A) and human (G) are comparable, each comprising an outer layer of trophectoderm (shown in green) surrounding a blastocyst cavity and an inner cells mass, which becomes divided into the epiblast (shown in blue) and the primitive endoderm (shown in yellow) lineages. In human embryos the primitive endoderm is often called the hypoblast. At early postimplantation stages, mouse (B-D) and human (H-J) embryos differ morphologically. At later stages human and mouse conceptuses appear to be morphologically more similar but the arrangements of the extraembryonic membranes differ (compare E, F to K, L). The mouse embryo is surrounded by the amnion, the visceral yolk sac and the parietal yolk sac (a rodent-specific membrane that usually ruptures after E12.5) but the chorion is incorporated into the placenta. In contrast, the human embryo is surrounded by an amnion and chorion but not the yolk sac. For simplicity, the placenta is shown in green (trophoblast) but it also contains extraembryonic mesoderm (shown in red elsewhere). See text for references.

Abbreviations: al, allantois; am, amnion; ch, chorion (exM+tr); ec, ectoplacental cone; emD, embryonic disk; emM, embryonic mesoderm; ep, epiblast; exEc, extraembryonic ectoderm; exM, extraembryonic mesoderm; H, Heuser’s membrane (from primitive endoderm); hyp, hypoblast (primitive endoderm); paEn, parietal endoderm; prEn, primitive endoderm; pys, parietal yolk sac (paEn + tr); tr, trophoblast (trophectoderm in A and G); uc, umbilical cord; vEn, visceral endoderm; vys, visceral yolk sac (vEn + tr); ys, yolk sac; ys1, primary yolk sac; ys2,secondary yolk sac.

References

Giurgea, I., Sanlaville, D., Fournet, J. C., Sempoux, C., Bellanne-Chantelot, C., Touati, G., Hubert, L., Groos, M. S., Brunelle, F., Rahier, J. et al. (2006). Congenital hyperinsulinism and mosaic abnormalities of the ploidy. *Journal of Medical Genetics* **43**, 248-254.

Kaiser-Rogers, K. A., McFadden, D. E., Livasy, C. A., Dansereau, J., Jiang, R., Knops, J. F., Lefebvre, L., Rao, K. W. and Robinson, W. P. (2006). Androgenetic/biparental mosaicism causes placental mesenchymal dysplasia. *Journal of Medical Genetics* **43**, 187-192.

Makrydimas, G., Sebire, N. J., Thornton, S. E., Zagorianakou, N., Lolis, D. and Fisher, R. A. (2002). Complete hydatidiform mole and normal live birth: a novel case of confined placental mosaicism: Case report. *Human Reproduction* **17**, 2459-2463.

Robinson, W. P., Lauzon, J. L., Innes, A. M., Lim, K., Arsovska, S. and McFadden, D. E. (2007). Origin and outcome of pregnancies affected by androgenetic/biparental chimerism. *Human Reproduction* **22**, 1114-1122.

Sheppard, S. E., Lalonde, E., Adzick, N. S., Beck, A. E., Bhatti, T., De Leon, D. D., Duffy, K. A., Ganguly, A., Hathaway, E., Ji, J. L. et al. (2019). Androgenetic chimerism as an etiology for Beckwith-Wiedemann syndrome: diagnosis and management. *Genetics in Medicine* **21**, 2644-2649.

Strain, L., Warner, J. P., Johnston, T. and Bonthron, D. T. (1995). A human parthenogenetic chimera. *Nature Genetics* **11**, 164-169.

Surti, U., Hill, L. M., Dunn, J., Prosen, T. and Hoffner, L. (2005). Twin pregnancy with a chimeric androgenetic and biparental placenta in one twin displaying placental mesenchymal dysplasia phenotype. *Prenatal Diagnosis* **25**, 1048-1056.
